# Interaction Metabolomics to Discover Synergists in Natural Product Mixtures

**DOI:** 10.1021/acs.jnatprod.2c00518

**Published:** 2023-04-13

**Authors:** Warren S. Vidar, Tim U. H. Baumeister, Lindsay K. Caesar, Joshua J. Kellogg, Daniel A. Todd, Roger G. Linington, Olav M. Kvalheim, Nadja B. Cech

**Affiliations:** † Department of Chemistry and Biochemistry, University of North Carolina at Greensboro, Greensboro, North Carolina 27402, United States; ‡ Department of Chemistry, Simon Fraser University, Burnaby V5A 156, BC, Canada; § Department of Chemistry and Biochemistry, James Madison University, Harrisonburg, Virginia 22807, United States; ∥ Department of Veterinary and Biomedical Sciences, Pennsylvania State University, University Park, Pennsylvania 16802, United States; ⊥ Department of Chemistry, University of Bergen, Bergen 5020, Norway

## Abstract

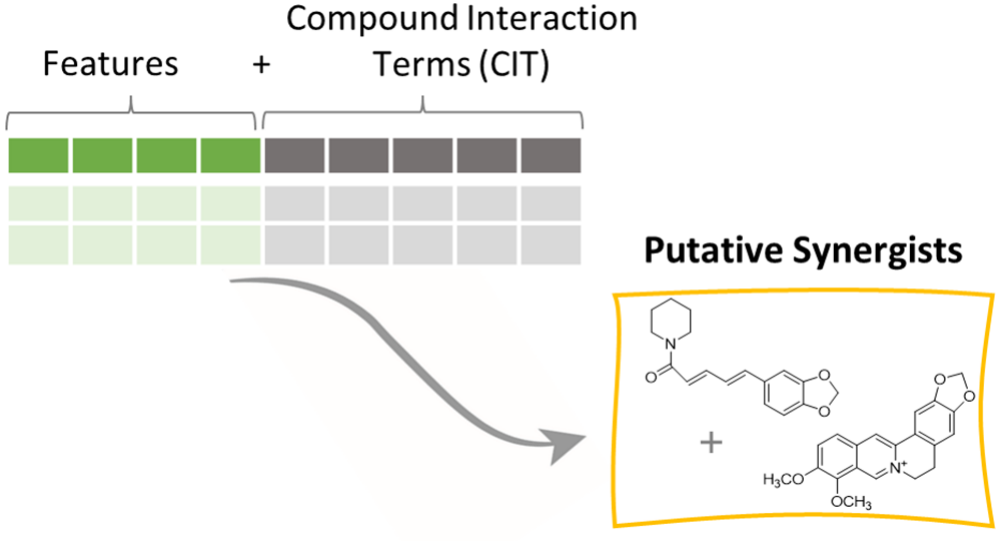

Mass spectrometry metabolomics has become increasingly popular as an integral aspect of studies to identify active compounds from natural product mixtures. Classical metabolomics data analysis approaches do not consider the possibility that interactions (such as synergy) could occur between mixture components. With this study, we developed “interaction metabolomics” to overcome this limitation. The innovation of interaction metabolomics is the inclusion of compound interaction terms (CITs), which are calculated as the product of the intensities of each pair of features (detected ions) in the data matrix. Herein, we tested the utility of interaction metabolomics by spiking known concentrations of an antimicrobial compound (berberine) and a synergist (piperine) into a set of inactive matrices. We measured the antimicrobial activity for each of the resulting mixtures against *Staphylococcus aureus* and analyzed the mixtures with liquid chromatography coupled to high-resolution mass spectrometry. When the data set was processed without CITs (classical metabolomics), statistical analysis yielded a pattern of false positives. However, interaction metabolomics correctly identified berberine and piperine as the compounds responsible for the synergistic activity. To further validate the interaction metabolomics approach, we prepared mixtures from extracts of goldenseal (*Hydrastis canadensis*) and habañero pepper (*Capsicum chinense*) and correctly correlated synergistic activity of these mixtures to the combined action of berberine and several capsaicinoids. Our results demonstrate the utility of a conceptually new approach for identifying synergists in mixtures that may be useful for applications in natural products research and other research areas that require comprehensive mixture analysis.

A central challenge in natural products research is the identification of biologically active compounds in complex mixtures.^1−5^ The gold standard approach toward accomplishing this task is bioassay-guided fractionation, wherein the mixture is subjected to successive stages of purification and biological evaluation until active compounds are identified. The value of bioassay-guided fractionation is evidenced by its history of success; many of the most therapeutically important natural products, molecules like artemisinin, Taxol, and penicillin, were discovered using this approach.^6,7^ What happens, however, when the activity of a mixture is not due to a single compound but to a mixture of compounds, which could act together synergistically, additively, or antagonistically? This question often arises in the study of botanical (herbal) medicines, which are employed therapeutically as mixtures rather than single molecules. Many proponents of the use of botanical medicines argue that they are effective by virtue of the combined action of multiple compounds.^8^ A number of studies point to the occurrence of synergistic biological effects in botanical extracts.^8−10^ In a few cases, the specific constituents or mechanisms responsible for this synergy have been identified. For example, artemisinin has been shown to be more potent in vivo against malaria when used as a complex tea than as an isolated molecule,^11^ and some plants contain both the antimicrobial alkaloid berberine and additional molecules that enhance the activity of berberine via efflux inhibition.^5^ However, the vast majority of natural product research focuses on the isolation of single active compounds, and there is a dearth of literature citing specific constituents that interact synergistically. It is possible that scenarios where multiple constituents in natural product mixtures exert meaningful combined biological activity are not, after all, very common. Alternately, perhaps our lack of knowledge about how combination effects arise is due to limitations in our ability to study them. Approaches that focus on the isolation and purification of single compounds may not fully explore the potential interactions that could contribute to the activity of mixtures.

There are five major requirements for an experimental design that enables identification of synergists in a mixture based on their association with biological activity: (1) Multiple mixtures must be evaluated for biological activity. (2) The active components must vary in concentration across the mixtures; otherwise, no new information is gained by testing multiple mixtures. (3) Two compounds that interact synergistically must be present at the correct range of concentrations to observe a synergistic effect. (4) The biological assay used must be appropriate for detection of synergy. (5) The method used to measure the presence and abundance of the mixture components must be able to detect the active constituents. Given requirements 1–5, there are multiple scenarios in which an analyst performing natural product drug discovery might fail to detect the presence of a synergist. The presence of a synergist will be missed if there are not enough measurements of biological activity, if the wrong biological activity is being measured, if the synergist and the active compound are not present in the same samples, if the synergist and the active concentration are not present at the correct concentrations to observe synergy, or if the analytical technique used for detection misses either the synergist or the active compound. Because of these inherent limitations, it will not be possible in a typical natural products drug discovery experiment to answer the question, “Are synergists present in this natural product extract?” The question that *can* be answered is the following question, “Could the activity that has been observed for a series of natural product mixtures be due to synergy between detectable compounds?” The primary objective of these studies was to develop a metabolomics data analysis approach that would address the second question.

The most widely used and validated approach for studying interactions between two biologically active molecules is the checkerboard assay.^1,3,10^ To conduct this assay, two compounds are tested in combination over a range of concentrations by 2-fold dilution. The data are then plotted in the form of an isobologram, which visually represents the changes in the dose–response behavior resulting from the combined effects of the two samples. If the dose–response behavior does not change when the two compounds are combined, the compounds are deemed to be non-interactive. Additivity results in a linear dose–response behavior, while synergy or antagonism is indicated by nonlinear changes in dose–response behavior.^10^ While checkerboard assays are most often employed to study combinations of pure compounds, they have also been employed using fractions to study synergy in botanical mixtures.^2,12^

The isobologram approach can be employed as a final validation step to confirm the types of interactions that occur between biologically active compounds.^13^ Practically speaking, however, it is not feasible to isolate every constituent from a biologically active natural product mixture and test activity in two-by-two combinations. In cases where the biological activity of a mixture may result from the combined effect of multiple compounds, some methodology is needed to help the analyst decide which mixture components to isolate and evaluate.

Several methods have previously been developed to identify synergists from complex natural product mixtures.^14,15^ One of these is synergy-directed fractionation,^3^ in which isolation is guided by measurements of the ability of one compound (or mixture of compounds^1^) to enhance the activity of a known active component of the mixture. With synergy-directed fractionation, it is possible to identify active compounds even if they do not possess activity alone. For example, this approach enabled the identification of flavonoids in *Hydrastis canadensis* (goldenseal) that have no inherent antimicrobial activity but enhance the activity of the alkaloid berberine.^1,3^ Building on synergy-directed fractionation, Caesar et al. developed an approach (called “Simplify”) to predict whether features identified in the liquid chromatography–mass spectrometry (LC-MS) data sets for complex mixtures interact synergistically, additively, or antagonistically. Simplify relies on the “activity index,” which is a measure of the ratio of the observed activity of a mixture to the activity that would be predicted based on concentration of a known active compound.^2^ The Simplify approach was employed to identify sugiol from the medicinal plant *Salvia miltiorrhiza*, and it was shown that sugiol synergistically enhances the antimicrobial activity of the alkaloid cryptotanshinone. Simplify is an effective strategy to identify constituents in a mixture that enhance the activity (synergistically or additively) of a known active compound. A limitation of this approach is that it requires a priori knowledge of the identity and concentration of this known active compound.

With the study described here, we set out to develop an approach to identify synergists that would be effective without prior knowledge of the concentration of an active constituent. We used untargeted LC-MS metabolomics as a central tool toward this goal. The application of LC-MS metabolomics to identify biologically active natural products relies on the integration of a “chemical” data set and a “biological” data set.^4,16,17^ The chemical data set consists of a set of features (ions detected by the mass spectrometer, each described by a characteristic mass to charge ratio, *m*/*z*, and retention time) and their associated abundance (peak height or peak area). The biological data set is a set of measurements that describe how each mixture perturbs a biological system (for example, inhibits cell growth, alters cell morphology, or reduces tumor size in an animal).

Several different data analysis approaches can be used to integrate these chemical and biological data sets. The most intuitive of these is to select the individual features in the chemical data set one-by-one and compare the abundance profile of each one to the biological activity of the samples. Such comparisons can be accomplished with univariate statistical methods such as Pearson correlation. In scenarios where more than one compound may be responsible for the activity of a natural product mixture, multivariate statistical approaches to data analysis are needed. For metabolomics data, multivariate latent-variable regression techniques such as partial least-squares (PLS) regression are particularly appropriate. PLS reduces the dimensionality of the regression model by linearly combining features. If used together with validation approaches, PLS addresses the problem of overfitting, which can occur in metabolomics data analysis because the number of observations of biological activity is often small compared with the number of variables (features) in the chemical data set. Such an “underdetermined” experimental design is more the rule than the exception in metabolomics studies.

Recent studies in natural products have described various workflows to predict active compounds from chemical and biological data sets. These workflows have been referred to with different terms, such as “compound activity mapping,”^18,19^ “bioactivity based molecular networking”,^20^ and “biochemometrics.”^4,21^ Compound activity mapping and bioactivity-based molecular networking successfully identified active compounds from natural product mixtures using Pearson correlations,^18−20^ while “biochemometrics” used PLS regression techniques for the identification of single active compounds^4^ and mixtures of natural products that act together additively.^22^

A limitation of data analysis workflows previously employed for natural products research^4,18−21^ is that they have operated under the assumption that the individual mixture components do not interact with each other. Our goal with this study was to develop and test a new “interaction metabolomics” data analysis approach for natural product discovery applicable in scenarios where mixture components interact to achieve biological effects. Toward this goal, we constructed an experimental system for which the observed biological activity was due to the interaction of known synergists. We measured antimicrobial activity against the bacterium *Staphylococcus aureus* in mixtures containing the antimicrobial alkaloid berberine^12,23^ and the synergist piperine.^1,24^ We also collected mass spectrometry metabolomics data for all of the mixtures and calculated a set of synthetic features from these data that we refer to as “compound interaction terms” (CITs). Each CIT represents a product of the peak areas of two features detected in the mixtures. Finally, we tested two data analysis workflows, one with these interaction terms included (“interaction metabolomics”) and one without the inclusion of the CITs (“classical metabolomics”) using both the simulated extracts (mixtures prepared from known constituents including berberine and piperine) and actual botanical extracts from the plants goldenseal (*Hydrastis canadensis*) and habañero pepper (*Capsicum chinense*). Our ultimate objective was to test whether CITs in the analysis workflow would enable the identification of the synergists that work in concert to exert antimicrobial effects.

## Results and Discussion

**Figure 1:**
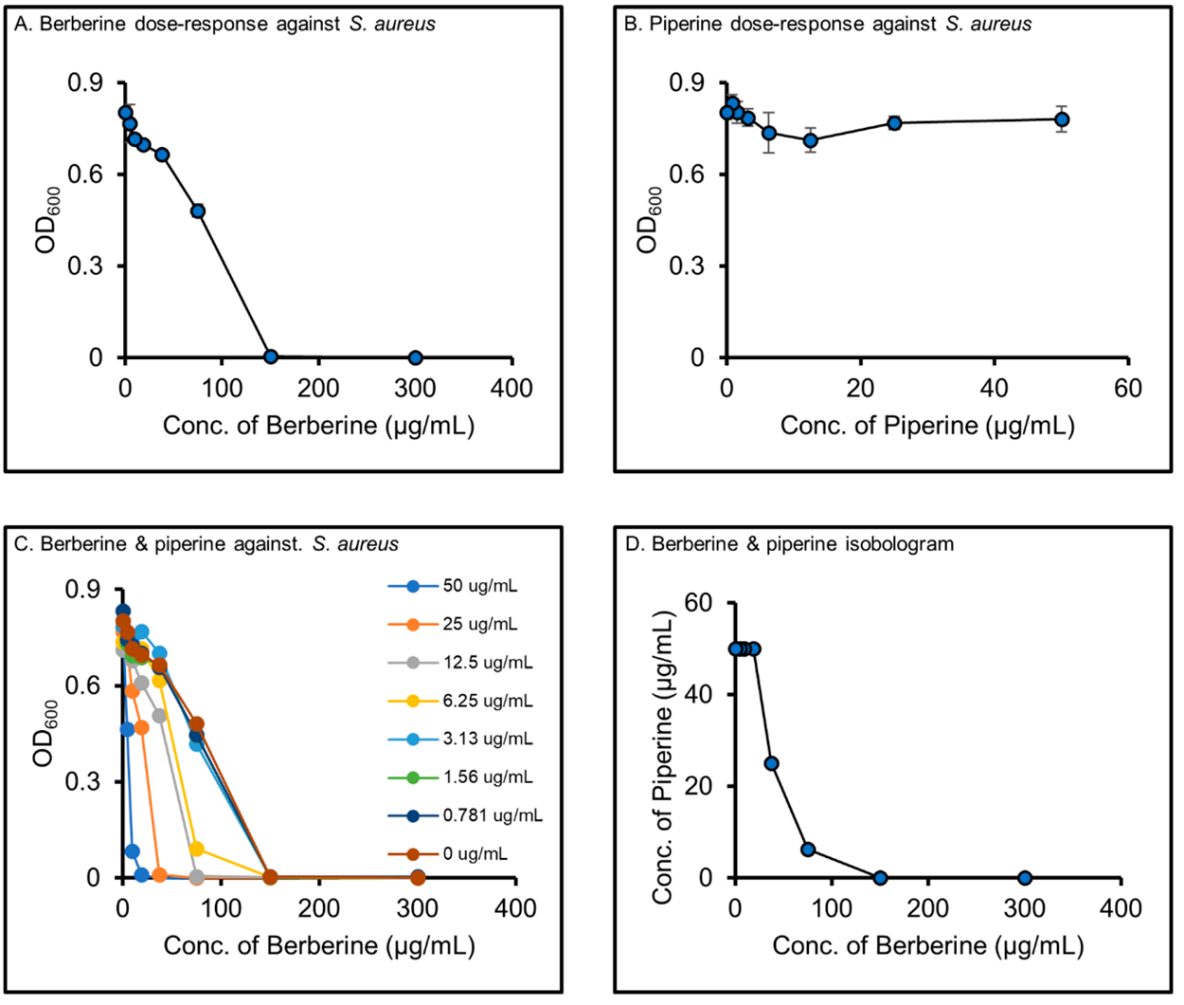
**Checkerboard assay results of berberine and piperine combinations against*****S. aureus.*** (A, B) The dose–response curves of berberine **(1)** and piperine **(2)** against *S. aureus*, respectively. (C) A dose–response curve of berberine combined with different concentrations of piperine (shown in different colors). Without added piperine, the MIC of berberine is 150 μg/mL. The addition of piperine reduces the MIC of berberine. For example, the MIC of berberine is 9.38 μg/mL in the presence of 50 μg/mL piperine. (D) A hyperbolic isobologram, indicating synergy. Additionally, the ∑FIC value (eq 1) of berberine in the presence of 50 μg/mL piperine is 0.19, signifying synergy.

Our first objective in this study was to create a scenario where all five of the requirements to observe synergy (see *Introduction*) were satisfied. Antimicrobial activity against *Staphylococcus aureus* was selected as the biological effect to be measured, and the number of mixtures necessary to distinguish a synergistic effect was determined using a modified factorial design.^25^ We selected a known antimicrobial (berberine, **1**(12)) and a known synergist (piperine, **2**(1,24)), both of which are detectable by LC-MS. We measured the dose–response behavior of these compounds alone and in combination (Figure 1) and determined the range of concentrations where direct antimicrobial activity of berberine (Table 1) or synergistic antimicrobial activity of berberine and piperine (Table 2) would be observed. We prepared a set of background matrices by chromatographically separating a mixture of inactive natural products (see Experimental Section for details on preparation), spiked berberine and piperine into these mixtures at the specified concentrations (Tables 1 and 2), and subjected each to analysis with LC-MS. The result of these experiments was a data set that we could use to develop and validate the interaction metabolomics approach to identify synergists. Notably, this data set, which is freely available, could also be employed by scientists seeking to benchmark other methodologies.^26^
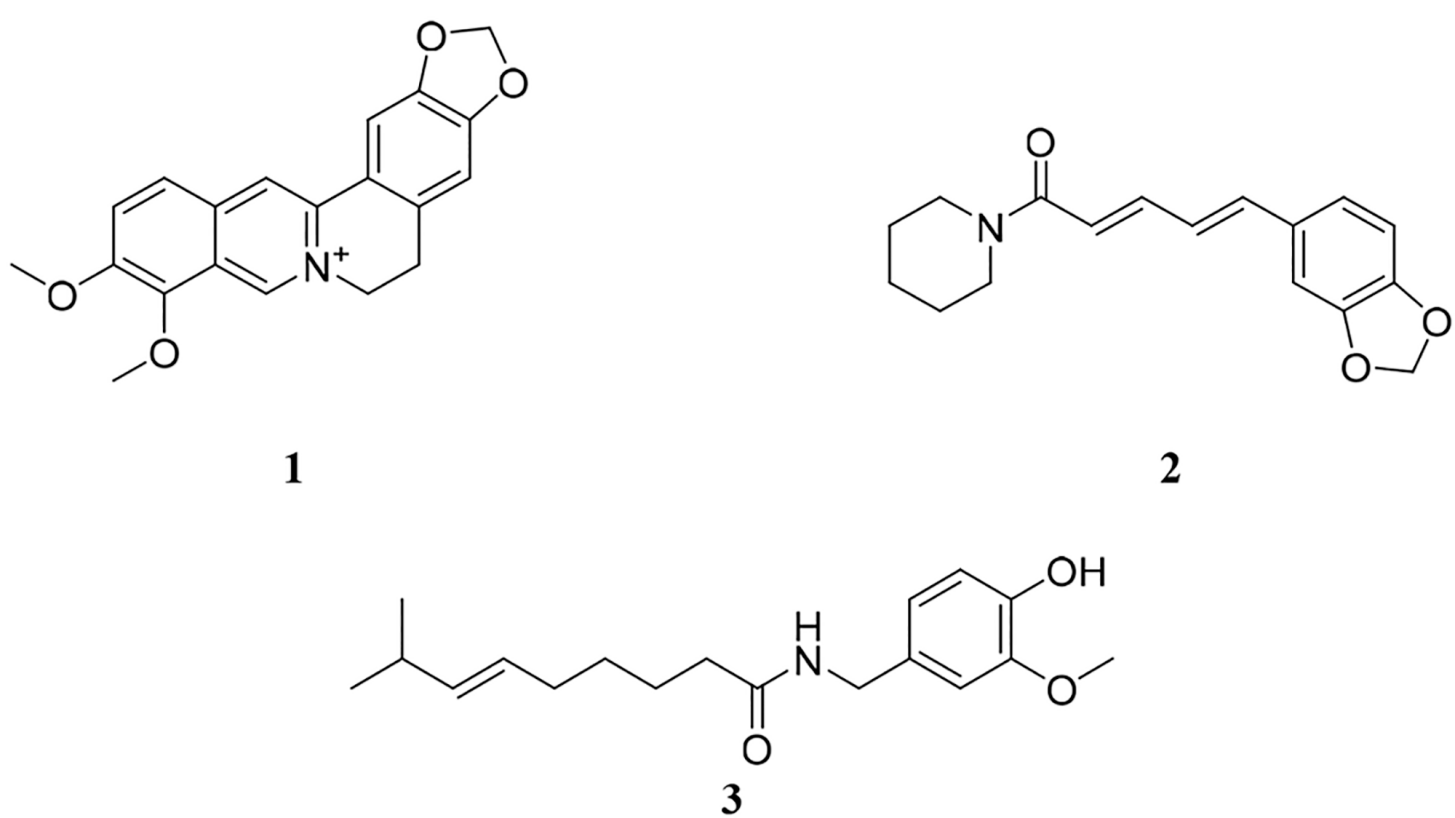


**Table 1:** Composition and Antimicrobial Activity of Berberine Spiked Fractions without Piperine^d^

**Sample**	**Berberine(μg/mL)**	**Piperine(μg/mL)**	**Fraction No.**^a^	**% inhibition(±s.d.)**^b^
M01	0	0	F01	5.4 (±8.9)
M02	100	0	F02	97.67 (±0.34)
M03	75	0	F03	97.50 (±0.64)
M04	64	0	F04	88.4 (±8.8)
M05	50	0	F05	29.0 (±4.5)
M06	32	0	F06	20.47 (±0.13)
M07	25	0	F07	16.9 (±2.1)
M08	16	0	F08	13.7 (±1.8)
Berberine (at 100 μg/mL)^c^				95.1 (±2.7)
Levofloxacin (positive control)^c^				99.61 (±0.22)

a Fraction indicates the pooled mixture of inactive compounds generated by flash chromatography separation of the simulated extract. Concentration is expressed as mass of the total mixture per well volume and does not indicate concentration of individual mixture components. Each mixture used a different fraction containing some subset of the compounds from the simulated extract. Each fraction was added at a concentration of 100 μg/mL, expressed as mass dried fraction per well volume in the antimicrobial assay.

b Antimicrobial activity is expressed as % inhibition relative to vehicle control ± standard deviation across triplicate wells.

c Levofloxacin and berberine were used as positive controls at 10 and 100 μg/mL, respectively.

d Concentrations indicate the assay concentrations used to evaluate % inhibition against *S. aureus*. Prior to analysis by LC-MS, samples were diluted 100-fold from the concentrations shown below to avoid saturation of instrument response.

**Table 2:** Composition and Antimicrobial Activity of Berberine and Piperine Spiked Fractions

**Sample**	**Berberine(μg/mL)**	**Piperine(μg/mL)**	**Fraction No.**^a^	**% inhibition(±s.d.)**^b^
M09	0	0	F01	7.4 (±3.1)
M10	32	0	F02	15.8 (±2.0)
M11	0	32	F03	8.7 (±1.6)
M12	32	32	F04	99.12 (±0.15)
M13	16	16	F05	31.2 (±2.8)
M14	8	8	F06	5.0 (±1.2)
M15	24	8	F07	13.7 (±3.7)
M16	8	24	F08	22.7 (±9.8)
M17	24	24	F09	64 (±12)
Berberine (at 32 μg/mL)^d^				17.5 (±2.7)
Piperine (at 32 μg/mL)^d^				3.2 (±1.4)
Levofloxacin (positive control)^c^				99.3 (±1.1)

a The same background matrices (fractions) used for mixtures 01–08 were used to prepare mixtures 09–16 here, plus another fraction for mixture 17. All fractions were tested at an assay concentration of 100 μg/mL.

b Antimicrobial activity is expressed as % inhibition of *S. aureus* growth relative to vehicle control ± standard deviation among triplicate wells.

c Levofloxacin was used as the positive control at 10 μg/mL.

d Berberine and piperine at 32 μg/mL were added as controls.

### Antimicrobial Activity of Berberine and Piperine is an Effective Model for Synergy

Berberine and piperine were selected for these studies as an antimicrobial and synergist, respectively. Berberine has been reported to inhibit the growth of *S. aureus*,^3,12,23^ while piperine had been reported to act as an efflux pump inhibitor.^1,24^ Berberine is a substrate to the NorA efflux pump in *S. aureus*, and piperine inhibits bacterial efflux, enhancing the antimicrobial activity of berberine without possessing any direct antimicrobial activity.^3,5,12^

To confirm the synergy between berberine and piperine, a checkerboard assay was conducted (Figure 1). Results show that berberine alone inhibits the growth of *S. aureus* with a minimum inhibitory concentration (MIC) of 150 μg/mL (446 μM) as previously reported^23^ (Figure 1A), while piperine alone does not show measurable antimicrobial activity (Figure 1B). As berberine is combined with increasing concentrations of piperine (Figure 1C), its MIC shifts to as low as 9.38 μg/mL (27.9 μM). An isobologram was plotted (Figure 1D), and using eq 1, the net fractional inhibitory concentration index of berberine and piperine (∑FIC) was calculated to be 0.19, which is ≤0.50, demonstrating synergy. Therefore, berberine and piperine possess synergistic antimicrobial activity against *S. aureus* under the conditions used in this study.
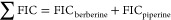
1where 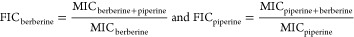


### Preparation, Characterization, and Antimicrobial Evaluation of the Spiked Fractions

**Figure 2:**
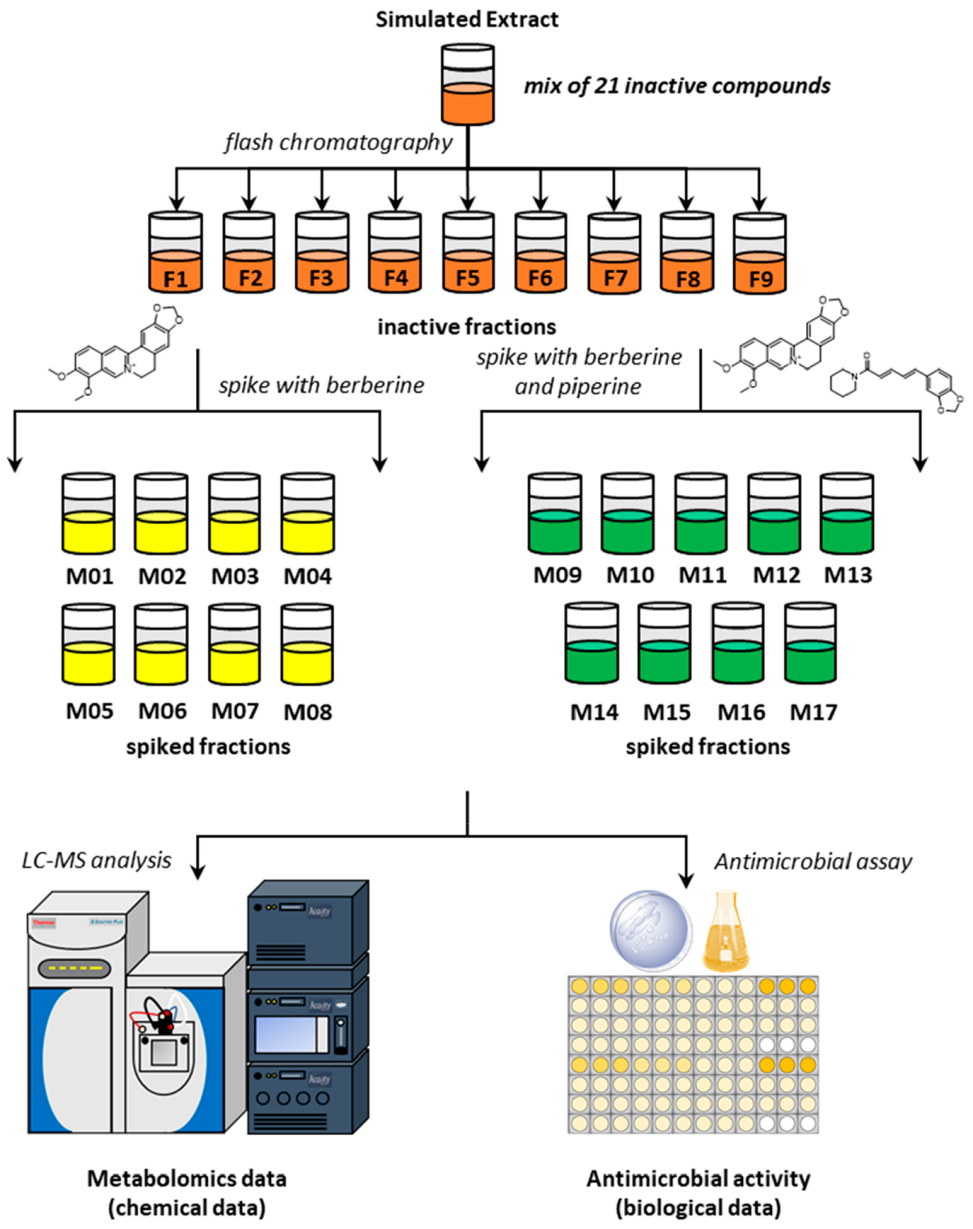
**Experimental workflow for preparation and analysis of spiked fractions.** A simulated extract was prepared by mixing 21 natural products that did not demonstrate antimicrobial activity against *S. aureus*, alone or in combination with berberine and piperine. This mixture was fractionated, and the pooled fractions were used as background matrices to create spiked fractions containing known amounts of berberine (antimicrobial) and piperine (synergist). Untargeted metabolomics data were collected with ultraperformance LC-MS to obtain a metabolomics data set. Antimicrobial activity was evaluated for all fractions against *S. aureus* to obtain a biological data set.

We employed a modified factorial design^25^ that enabled a demonstration of the interaction effects between berberine and piperine using a smaller number of mixtures (total of 9) than is typically employed to collect an isobologram.^13^ To conduct this experiment, we prepared nine mixtures with known concentrations of berberine and piperine, each with an inactive background matrix. The inactive matrices were created by using flash chromatographic separation of a simulated natural product extract (Figure 2). Thus, each fraction had varying levels of the inactive constituents similar to what would be obtained by a typical natural products isolation experiment. We controlled the levels of berberine and piperine by spiking them into the mixtures after chromatographic separation because we wished to create a test system where synergy would certainly occur.

To prepare the simulated extract, we tested the antimicrobial activity of a series of 42 commercially available natural products (Table S1) to select those that fulfilled the following two selection criteria: (1) did not inhibit *S. aureus* growth by >20% (eq 2) at assay concentration of 100 μM and (2) did not enhance the antimicrobial activity of berberine or piperine. A subset (22) of the compounds tested fit criterion 1 (Table S1). When these compounds were tested in combination with berberine (32 μg/mL or 95 μM) or piperine (32 μg/mL or 112 μM), none enhanced the activity of piperine, and only capsaicin (**3**) enhanced the antimicrobial activity of berberine (Table S2). The observed enhancement of berberine activity by capsaicin was consistent with a previous report of capsaicin being an inhibitor of the NorA efflux pump of *S. aureus*.^27^ Therefore, capsaicin was not included in the simulated mixture, reducing the number of compounds to 21. Finally, the components were all analyzed with LC-MS, and all except stigmasterol and β-sitosterol were detected in extracted ion chromatograms and by peak picking above the threshold of 1 × 10^5^ using MZmine 2.53^28^ (Table S3).

Flash chromatography was employed to separate the simulated extract into ten pooled fractions. From these, the nine pooled fractions that yielded sufficient material for the experiments were used to create a series of fractions spiked with berberine alone (M01-M08) (Table 1) or with berberine and piperine (M09-M17) (Table 2). Different concentrations of berberine were used between the two sets of mixtures, because berberine antimicrobial activity was expected to saturate at higher concentrations in the mixtures without piperine. The background matrices used to create the spiked fractions were prepared with a final assay concentration of 100 μg/mL (expressed as the mass of dried material per mL of assay volume). As in a realistic natural products isolation experiment, the concentrations of individual background matrix components in these mixtures were not known.

To obtain the “biological data set,” antimicrobial activity was measured for the pooled fractions alone (Figure S1) and for the fractions spiked with berberine (Figure S2A) or berberine and piperine (Figure S2B). As expected, the pooled fractions alone (without berberine or piperine) demonstrated less than 20% growth inhibition of *S. aureus* (Figure S1). Spiking the fractions with berberine alone (M01-M08) caused dose-dependent inhibition of bacterial growth (Figure S2A). The mixtures spiked with berberine and piperine (Figure S2B) also suppressed bacterial growth, with the highest activity observed for mixture 12 (M12). Synergistic antimicrobial activity was observed for several of the mixtures (Figure S2B).

LC-MS analysis was conducted on all the spiked fractions to obtain the “metabolomics data set” (Figures S3–S6). Berberine was readily detectable in M01-M08 (Figures S3 and S4), while berberine and piperine were detectable in M09-F17 (Figures S5 and S6). Berberine and piperine were identified by their characteristic mass spectral data (Figure S7). The mass spectrum for berberine (Figure S7A), which has an inherent positive charge, is characterized by a single peak representing the M^+^ ion. Piperine is detected as a series of five features (Figure S7B), including the protonated species [M + H]^+^, sodiated species [M + Na]^+^, proton bound dimer [2M+H]^+^, sodium bound dimer [2M+Na]^+^, and a sodiated acetonitrile cluster [M+ACN+Na]^+^.

Analysis of standards indicated that all of the inactive matrix compounds were detectable by LC-MS except stigmasterol and β-sitosterol (Table S3), for a total of 19 detectable compounds. Similarly, and as would be expected, each of the inactive matrix compounds was also detectable in at least one of the spiked fractions (Table S4). Of the 19 components detected in the original data set (Table S4), seven (naringin, chlorogenic acid, tropine, *p*-octopamine, vanillic acid, and theobromine) were removed in the data filtering step that required variation in abundance across the mixtures (Table S5). Thus, the final metabolomics data sets included features (Table S6) from berberine, piperine, and 12 inactive compounds.

### Conceptual Demonstration of the Compound Interaction Term with Spiked Fractions

**Figure 3:**
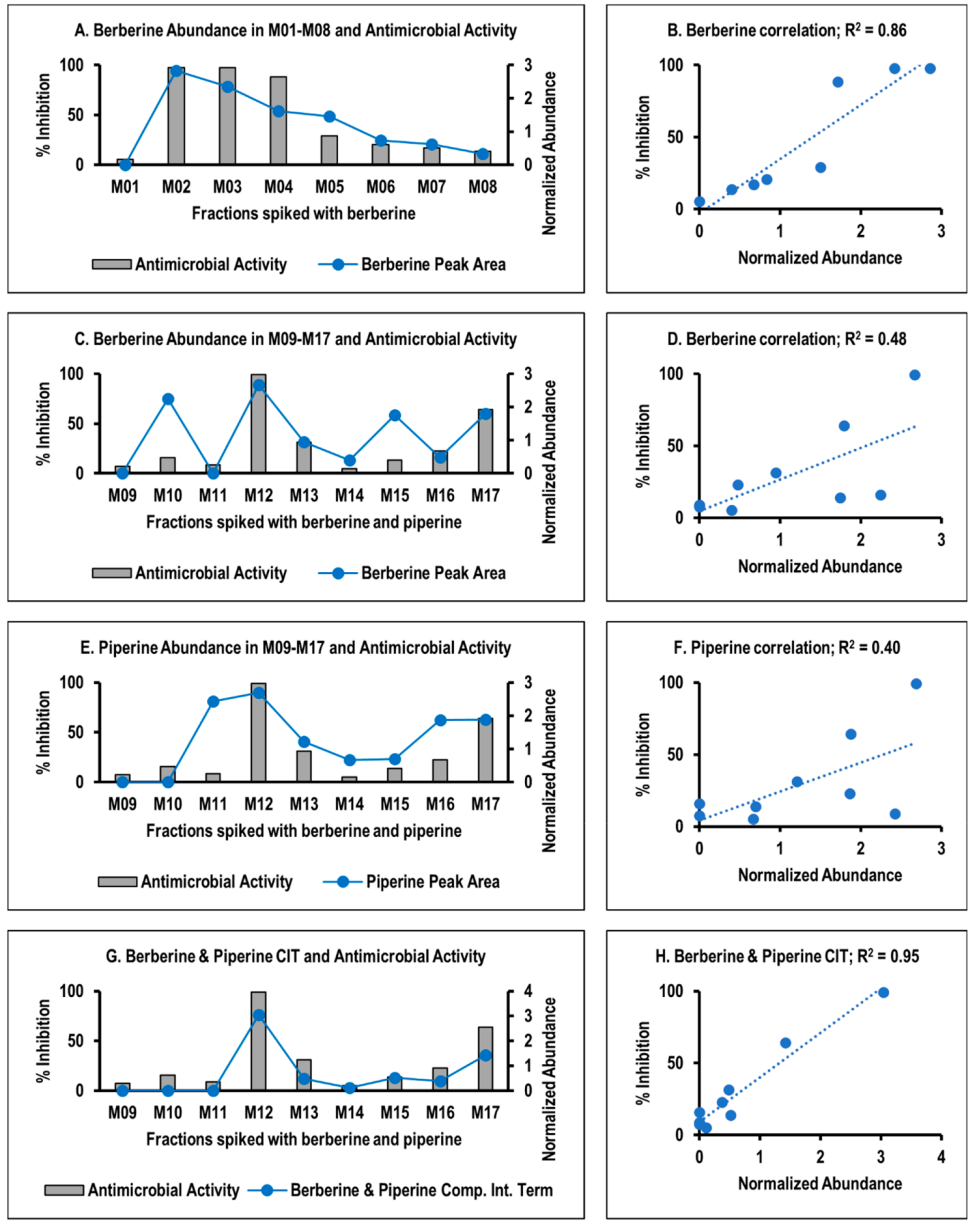
Comparison of biological and chemical data demonstrates the utility of the CIT. The antimicrobial activity (% inhibition of *S. aureus* growth) was strongly correlated with standardized abundance for the fractions spiked with berberine (M01-M08, panels A and B). For the mixtures spiked with berberine and piperine (M09-M17), neither berberine (C) nor piperine (E) abundance tracks with biological activity. This lack of correlation is shown with poor linearity of regression plots for % inhibition vs peak area of berberine (D) and piperine (F). The CIT obtained by multiplying the peak area for piperine with the peak area for berberine (eq 3) tracks with biological activity for the berberine-piperine mixtures (G). The linear relationship between the % inhibition and the CIT is demonstrated in panel H. To generate these plots, piperine abundance was measured as the peak area of the selected ion for the [M + H]^+^ ion of piperine detected at *m*/*z* 286.1426, while the peak area of berberine was measured as the peak area of the M^+^ ion detected at *m*/*z* 336.1217. The peak areas for these ions were standardized, as shown by eq 5.

To demonstrate the concept of the CIT (eqs 3 and 4), we examined the data obtained from the chemical and biological analyses of the spiked fractions by selecting major features associated with berberine (M^+^) and piperine ([M + H]^+^) and comparing their standardized abundance (eq 5) with the measured biological activity of the fractions (Figure 3). For the fractions spiked with just berberine (M01-M08, Figure 3A), biological activity is correlated with the peak area of berberine. For the fractions that contain both berberine and piperine (M09-M17), neither the peak area of the berberine feature (Figure 3C,D) nor the peak area of the piperine feature (Figure 3E,F) is strongly correlated to biological activity. This is expected given that the antimicrobial activity of the mixtures results from the combined (synergistic) activity of berberine and piperine. To obtain a value that correlated with activity in the berberine-piperine mixtures, a CIT (eq 3) was obtained by multiplying the peak area of one berberine feature (M^+^) by the peak area of one piperine feature ([M + H]^+^). This CIT tracks closely with antimicrobial activity (Figure 3G), and the relationship is linear (*R*^2^ value of 0.95; Figure 3H).

### Comparison of Classical and Interaction Metabolomics Workflows Using Spiked Fractions

**Figure 4:**
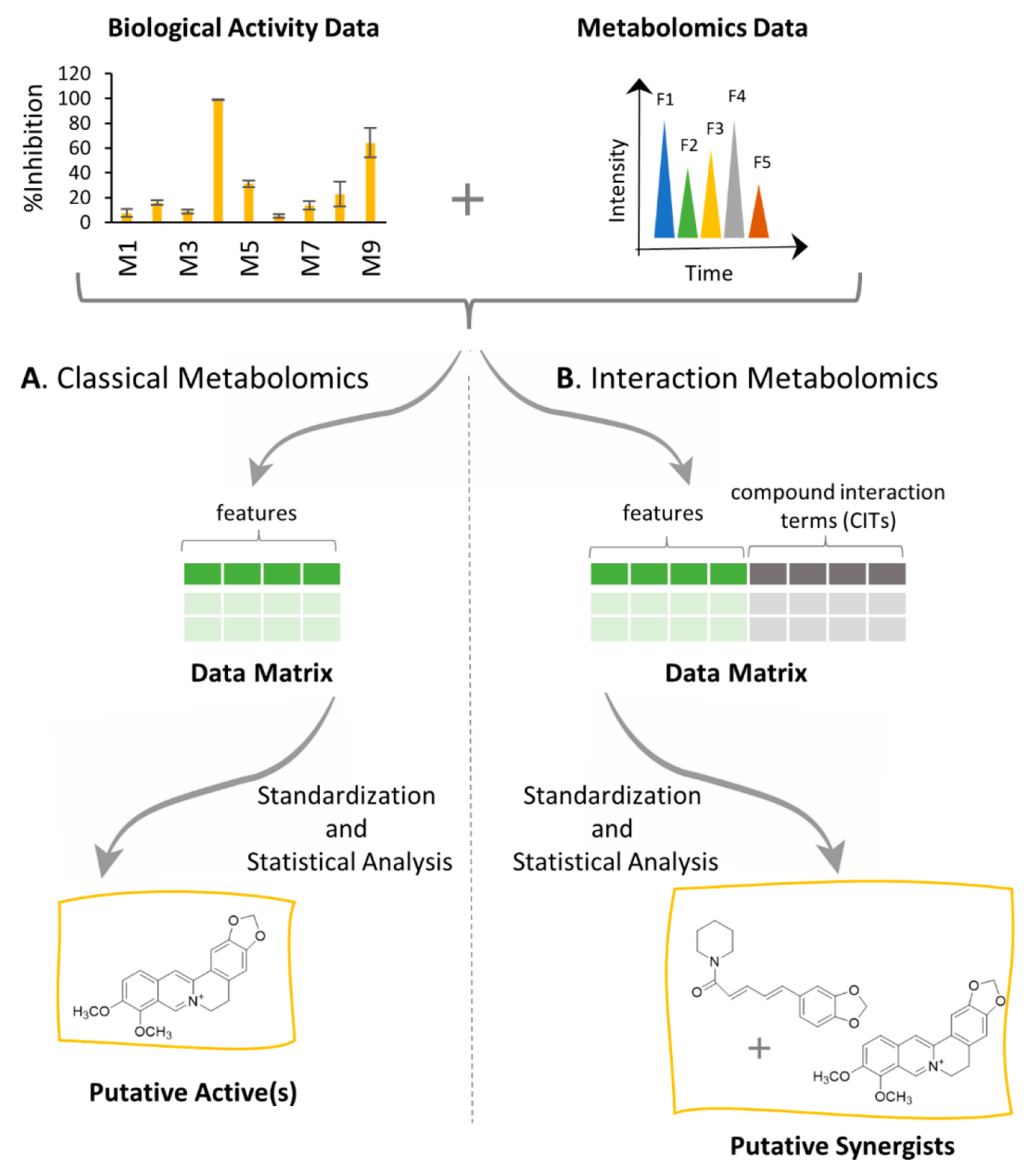
Two possible metabolomics workflows for data analysis: (A) classical metabolomics and (B) interaction metabolomics. The two workflows both start with a biological data set and a metabolomics data set (in this case LC-MS data obtained by analysis each of the mixtures individually). The values from these biological and metabolomics data sets are compiled in one of two data matrices (Figure S8). The data matrix for the interaction workflow differs in the inclusion of the CITs. The values in the data matrices are then standardized (eq 5), and multivariate statistical analysis is conducted, resulting in the prediction of putative antimicrobials (classical metabolomics) or putative synergists (interaction metabolomics).

While a univariate approach, as depicted in Figure 3, is useful for demonstrating the CIT idea, a multivariate statistical approach is more appropriate when untargeted metabolomics data sets containing many features are analyzed. Here we analyzed the data with multivariate statistics in a “classical metabolomics” workflow (Figure 4A) and an “interaction metabolomics” workflow (Figure 4B). The difference between the two workflows is the data matrix (Figure S8) used for analysis. The classical workflow was conducted with a matrix containing biological activities and standardized feature intensities for each of the mixtures. In the workflow allowing interactions, the matrix was expanded to include standardized CITs for each feature pair in the data set. Both data sets used in these calculations are freely available.^26^

### Spiked Fractions Demonstrate the Importance of Data Filtering for Interaction Metabolomics

Prior to the data filtering steps used for reducing the number of features (see *Experimental Section*), the number of features detected in the berberine spiked mixtures (M01-M08) was 2894 (Table S7). By eq 4, 2894 features would yield 4 186 171 CITs, which would result in a data matrix with 4 189 065 variables (4 186 171 CITs + 2894 features), far too many to create a meaningful PLS model. To reduce the number of features to a manageable number for an interaction metabolomics workflow, we removed features that were present in the blank and any features for which the relative standard deviation of peak area across triplicate analyses was >35%. This reduced the total number of features in the berberine spiked mixtures to 232. We then further filtered the data by removing any features for which peak area did not vary by more than 0.01% across all samples. The rationale for this filtering step was the assumption that, if biological activity varies across the samples, abundance of the compounds responsible for activity should also vary. The final filtered data set had 26 features, and the data matrix including features and CITs had a manageable total of 207 variables (Table S7). Note, 207 is lower than the predicted total number of variables (26 features + 325 CITs = 351 variables) because any feature with peak area of zero will result in a CIT of zero. Similarly, the total number of features in the berberine and piperine spiked mixtures was 33 after all data filtering steps (Table S7), and the final CIT data matrix contained a total of 462 variables.

### Comparison of Putative Active Constituents in the Spiked Fractions Predicted by Classical Metabolomics and Interaction Metabolomics

For both classical metabolomics and interaction metabolomics (Figure 4), PLS was used to determine which features in the chemical data set for the spiked fractions were most strongly associated with biological activity, an approach often referred to as “biochemometrics”. As with previous biochemometrics studies,^1,2,4,16,17,22,29^ we employed the selectivity ratio as a measure of which mixture components associate with biological activity. The selectivity ratio is obtained by dividing the variance explained by the target projection component for a given feature by the residual variance (see *Experimental Section*, Figure S9). Because the association between biological activity and ion abundance indicated by selectivity ratios is purely correlative, false correlations may occur. Thus, predictions of biological activity based on the selectivity ratio are deemed “putative” and would typically be followed up by a validation experiment testing activity of the isolated compounds. For this study, the biological activity of the mixture components was known a priori, so the validity of the predictions from the multivariate statistical model could easily be tested. We predicted that the application of classical metabolomics (Figure 4A) to the data for mixtures 01–08 would assign a high selectivity ratio to the features associated with berberine and that application of interaction metabolomics (Figure 4B) to mixtures 09–17 would assign high selectivity ratios to the CITs associated with berberine and piperine.

**Figure 5:**
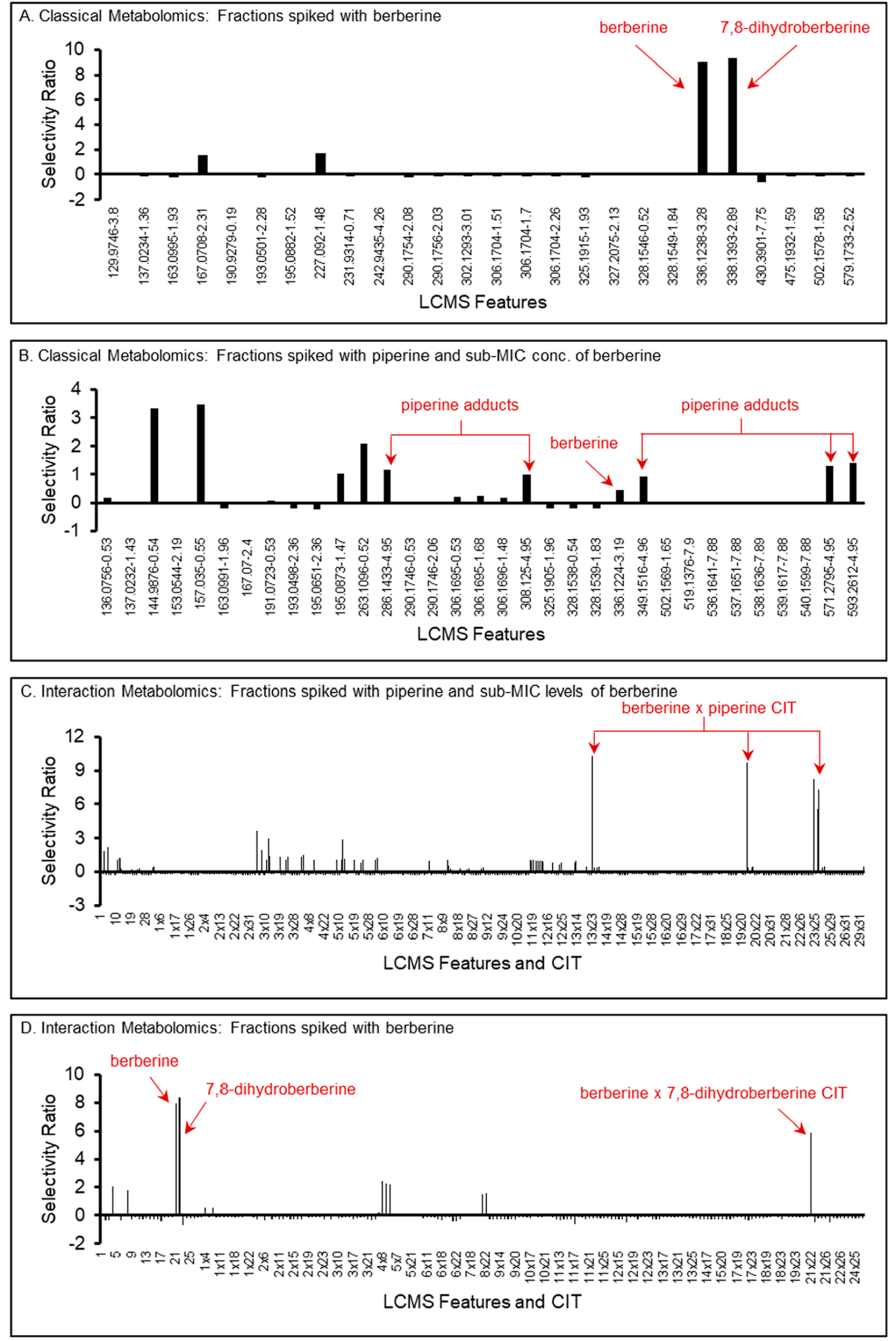
Comparison of selectivity ratio plots for the spiked fractions using classical metabolomics (A, B) and interaction metabolomics (C, D). The five CITs in (C) are the products of berberine and the five piperine adducts: protonated species [M + H]^+^, sodiated species [M + Na]^+^, proton bound dimer [2M+H]^+^, sodium bound dimer [2M+Na]^+^, and a sodiated acetonitrile cluster [M+ACN+Na]^+^. Specifically, in Panel C, **13 × 23 =** piperine [M + H]^+^ × berberine M^+^, **19 × 23 =** piperine [M + Na]^+^ × berberine M^+^, **23 × 24 =** berberine M^+^ × piperine [M+ACN+Na]^+^, **23 × 32 =** berberine M^+^ × piperine [2M+H]^+^, and **23 × 33 =** berberine M^+^ × piperine [2M+Na]^+^. In panel D, **21** × **22** = berberine M^+^ × [M + H]^+^ of putative 7,8-dihydroberberine. The total number of features in the data set were 17, 32, 528, and 153 for panels A, B, C, and D, respectively. Prior to multivariate statistical analysis, all metabolomics data were filtered based on the requirement that they demonstrate consistent peak area across all replicate analyses and the requirement that the feature area vary by >0.01% across all samples in the mixture (see *Experimental Section*).

The effectiveness of the two workflows (Figure 4) for predicting active mixture components is compared in Figure 5. The plots in this figure show the magnitude of the selectivity ratio on the *y*-axis calculated for each explanatory variable (feature or CIT) on the *x*-axis. Note that the selectivity ratio plot includes features detected across all of the samples included in the analysis and as such are a composite view that differs from the more common way of viewing LC-MS data for each mixture individually.

There is no absolute cutoff value for what qualifies as a “relevant” selectivity ratio. The magnitude of the selectivity ratios obtained in a given analysis will vary depending on the unique characteristics of the data set being inspected. It is most useful to think of selectivity ratios as a ranking tool. Compounds associated with features that have high selectivity ratios are associated with biological activity and can be prioritized for isolation and further testing. In this case, since we knew for each data set which compounds were active, we considered the selectivity ratio analysis to be successful when the features or interaction terms with highest selectivity ratios corresponded to those known active compounds.

When the classical metabolomics workflow (Figure 4A) was applied to the mixtures spiked only with berberine (M01-M08), one of the two features with the highest selectivity ratio corresponded to the M^+^ ion for berberine (*m*/*z* 336.1238, 3.28 min) (Figure 5A). The second of the two features with the highest selectivity ratio (*m*/*z* 338.1393, 2.89 min) was tentatively assigned to the molecule 7,8-dihydroberberine. Inspection of the berberine standard by LC-MS (Figure S10) indicated the presence of the 7,8-dihydroberberine feature (at 21 times lower intensity than berberine); thus, it appears that 7,8-dihydroberberine is a contaminant of the “pure” berberine. The predicted activity of 7,8-dihydroberberine highlights one limitation of the biochemometrics approach for predicting active mixture components; any feature that correlates with activity will be predicted to be active, but the prediction is purely correlative and must be verified by testing of the compound in isolation. Previous literature has reported that the antimicrobial activity of 7,8-dihydroberberine is similar to that of berberine,^30^ suggesting that this compound may contribute to the overall activity of this fraction (not evaluated). Several other features are given nonzero selectivity ratios in this analysis (Figure 5A). These are likely false positives but would be deprioritized for isolation given that their selectivity ratio values are small relative to the features associated with berberine and the putative 7,8-dihydroberberine.

One might expect (as we did at the onset of this study) that PLS analysis of a system where activity is due to synergy would generate a poor model because compound interaction would be overlooked. Instead, PLS modeling created what would be considered a “good” fit of the data for the mixtures spiked with piperine and berberine (M09-M17, Figure 5B). Despite the good fit of the model to the experimental data, we know it is incorrect. Features that correspond to known inactive components of the fractions were incorrectly assigned larger selectivity ratios than berberine or piperine (Figure 5B). This appears to be a case of confounding. Thus, the data in Figure 5B demonstrate a crucial limitation of classical PLS modeling of metabolomics data. If the observed biological effect is due to synergy, a model that appears to be of high quality can be obtained even though the association pattern between activity and analytes is wrong due to the missing interaction term.

When the new interaction metabolomics approach (Figure 4B) was applied to the data sets containing CITs, the resulting selectivity ratio plot (Figure 5C) showed the correct prediction of active constituents. From the 462 features and interaction terms in the data set (Table S7), only five had high selectivity ratios. All of these high selectivity ratio features correspond to the CITs for piperine features combined with berberine features. Therefore, the problem of confounding demonstrated in Figure 5C is resolved when a data set that includes interaction terms is utilized. Importantly, multiple features are detected in this selectivity ratio plot (Figure 5C) because the single piperine molecule forms five different cluster ions in the source of the mass spectrometer (Figure S7). Thus, although five CITs are found, they are redundant in that each corresponds to the berberine feature area multiplied by the area of a feature representing a different ion formed by the LC-MS analysis of piperine.

Because we knew which components in the mixtures were active, it was possible in this study to diagnose the problem of a confounded model (Figure 5B) and correct it with the inclusion of CITs (Figure 5C). However, an analyst working with a system of unknown composition could waste a great deal of time pursuing putative active compounds with high selectivity ratios in Figure 5B, only to discover that none of them are active. Both of the models in Figure 5B,C were of almost similar quality, having root-mean-squared error of prediction (RMSEP) values of 14.02 and 11.09, respectively, and *R*^2^*Y* values of 0.907 and 0.985, respectively (Table S8). Thus, it is not possible to diagnose the confounding problem observed in Figure 5B based on the model parameters alone.

A possible solution to prevent a false model such as that shown in Figure 5B would be to include interaction terms in any metabolomics data set, preemptively considering the possibility that activity is due to compound interactions. To test the effectiveness of such an approach, we applied interaction metabolomics to the data set for the mixtures containing only berberine (Figure 5D), where we knew that activity was not due to compound interactions. A concern prior to carrying out this data analysis was that the inclusion of CITs might introduce additional false correlations. However, even with the CITs included, selectivity ratio analysis of the berberine spiked mixtures predicted berberine and putative 7,8-dihydroberberine to be the active constituents (Figure 5D), similar to the conclusion for the data matrix without CITs (Figure 5A). These results suggest that interaction metabolomics might be an applicable approach, even when compounds in the mixture do not interact.

Notably, and as would be expected, the CIT for the berberine × 7,8-dihydroberberine produced a high selectivity ratio in Figure 5D. Thus, the inclusion of CITs might enable the identification of combinations of compounds that interact additively, a concept that would be a good topic of future exploration. Importantly, however, the data in Figure 5D demonstrate that a CIT with high selectivity ratio could be indicative of either synergy, additivity, or a false correlation. These results underscore the importance of additional validation for any predictions made based on a statistical comparison of chemical and biological data sets.

### Evaluation of Interaction Metabolomics Using Complex Botanical Extracts

To further evaluate interaction metabolomics, we sought to extend the approach to a model system of complex botanical extracts. The data in Table S2 demonstrate that capsaicin (3) and berberine (1) possess synergistic antimicrobial activity; thus, we elected to test interaction metabolomics using a botanical extract from goldenseal (*Hydrastis canadensis*), a plant known to produce berberine,^3,5,12^ and habañero pepper (*Capsicum chinense*), a plant known to produce capsaicin.^31^

**Figure 6:**
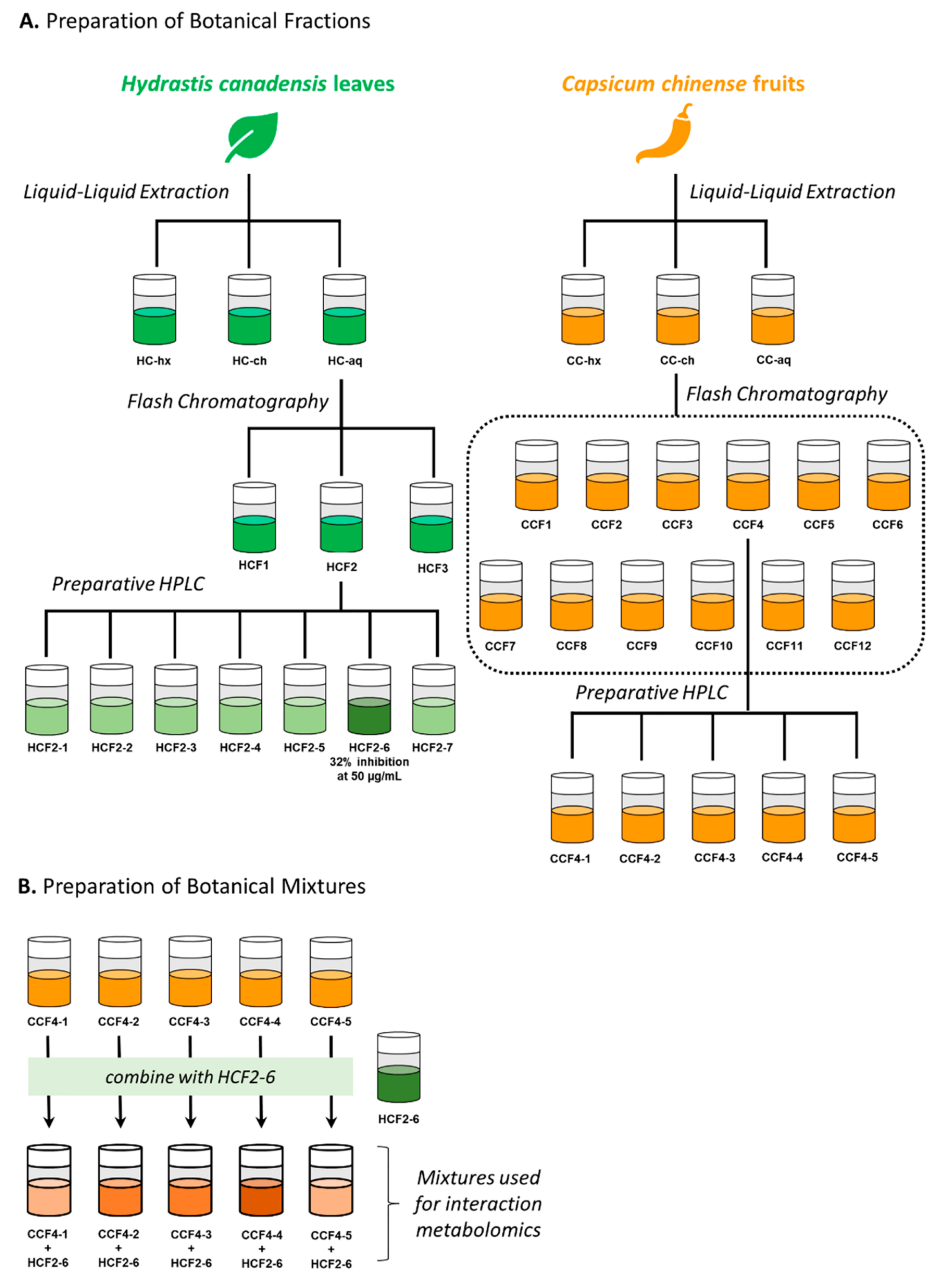
Workflow used to prepare fractions from *Hydrastis canadensis* and *Capsicum chinense* (A) and procedure for mixing fractions (B). To prepare the subfractions of *H. canadensis*, the *H. canadensis* partition highest in berberine (HC-*aq*) was separated into three fractions with flash chromatography. The fraction with highest berberine content (HCF2) was further fractionated into six subfractions using preparative HPLC, and of these, HCF2–6 had the strongest antimicrobial activity (32% inhibition of *S. aureus* at 50 μg/mL, Figure S11A) and the highest content of berberine (Figure S12). To prepare the subfractions of *C. chinense*, the partition with highest content of capsaicin (CC-*ch*) was selected and separated into 12 fractions using flash chromatography. The fraction with the highest capsaicin content (CCF4) was then fractioned into five subfractions, CCF4-1, CCF4-2, CCF4-3, CCF-4, and CCF-5, using preparative HPLC (Figure S13). Mixtures were prepared by combining a sub-MIC concentration (50 μg/mL) of the most strongly antimicrobial subfraction from *Hydrastis canadensis* (HCF2–6) with all of the subfractions from the *C. chinense* (CCF4-1 through CCF4-5), each at 100 μg/mL.

Successful application of interaction metabolomics to botanical extracts required that we devise a strategy to ensure that the synergistic constituents (berberine and capsaicin) would end up in the same mixtures, enabling synergy to be observed. One option to accomplish this would have been to mix extracts of the two botanicals together and then separate the resulting mixture, hoping that berberine and capsaicin might coelute in some of the fractions. We opted instead to increase the likelihood that synergists co-occur in mixtures by intentionally recombining fractions after chromatographic separation, as has been done previously.^12,22,32^ Extracts were prepared separately from *H. canadensis* and *C. chinense*, and these extracts were each subjected to partitioning and fractionation (Figure 6A).

In previous studies, we determined that biochemometric analysis is most successful using extracts that have been subjected to two stages of chromatographic separation.^1,2^ Thus, for this study, we prepared extracts of *H. canadensis* and *C. chinense*, partitioned them with liquid–liquid partitioning, and separated them first with normal phase flash chromatography and then by reversed-phase preparative high-performance liquid chromatography (HPLC) (Figure 6A). All of these fractions were also evaluated for antimicrobial activity and were only weakly antimicrobial (Figure S11). To create mixtures for interaction metabolomics analysis (Figure 6B), we selected the single subfraction of *H. canadensis* with the most potent antimicrobial activity (HCF2–6, Figure S11A) and combined it with each of the *C. chinensis* subfractions. We chose a single high concentration of the *C. chinensis* fraction (100 μg/mL) to maximize the likelihood of observing an interaction and a sub-MIC concentration of the *H. canadensis* fraction (50 μg/mL) to avoid saturating the antimicrobial activity.

Following the interaction metabolomics workflow in Figure 4, the mixtures prepared from *H. canadensis* were analyzed by ultraperformance liquid chromatography–mass spectrometry (UPLC-MS) (Figure S14) to create a metabolomics data set and tested for antimicrobial activity (Figure S15) to create a biological activity data set. The metabolomics data set after filtering consisted of 74 features. The data matrix with CITs contained a total of 132 features and interaction terms. A concern we had when embarking on the studies with botanical extracts was that the mixtures would be too complex to be effectively interrogated using interaction metabolomics, especially given that the data matrix is expanded by the addition of CITs. Contrary to this concern, the data matrix for the botanical extract experiment proved to be less complex than the data matrix for the artificial mixtures (462 features and CITs). Notably, however, two rounds of fractionation were conducted to simplify the botanical extracts. The number of features would have been much higher without these fractionation steps.

**Figure 7:**
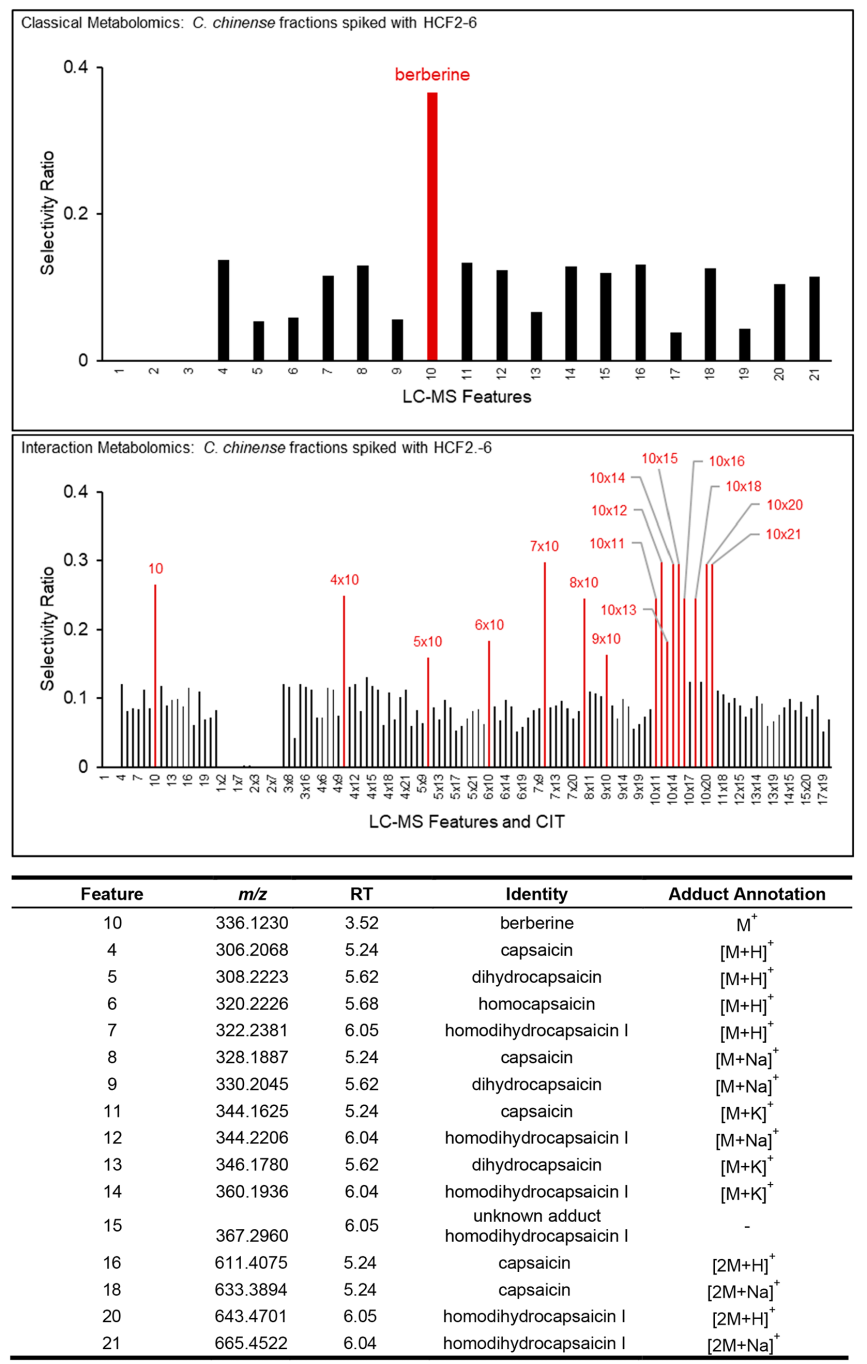
Selectivity ratio plot generated with classical metabolomics (A) and interaction metabolomics (B) using the metabolomics data and antimicrobial data obtained from the *H. canadensis* and *C. chinense* mixtures prepared as shown in Figure 6. For classical metabolomics (A) the predominant feature in the selectivity ratio plot corresponds to protonated berberine (the M^+^ ion with *m*/*z* 336.1230). With interaction metabolomics (B), synergists are also detected. Of the 17 high selectivity ratio features or CITs highlighted in red, 16 could be assigned either to berberine alone or to berberine interacting with one of four putative capsaicinoids.

Selectivity ratio of the *H. canadensis/C. chinense* mixtures using classical metabolomics and interaction metabolomics both yielded suitable PLS models (Table S10). However, classical metabolomics (Figure 7A) highlighted a single feature in the data set, which corresponded to berberine (M^+^, *m*/*z* 336.1230). Interaction metabolomics analysis of the same data (Figure 7B) yielded a total of 16 features or CITs with high selectivity ratios. These were assigned either to berberine alone (feature 10, *m*/*z* 336.1230) or to berberine interacting with features detected in the *C. chinensis* extract. As expected, the interaction terms the berberine feature, with all four major features detected in the mass spectrum of capsaicin (Figure S16A), yielded high selectivity ratios (Figure 7). Several additional features with high selectivity ratio were assigned to berberine interacting with three other capsaicinoids (Figure 7), putatively identified as dihydrocapsaicin (Figure S16B), homodihydrocapsaicin I (Figure S16C), and homocapsaicin (Figure S16D).

Overall, the data in Figure 7 demonstrate that interaction metabolomics can be employed to detect synergists that are missed with classical metabolomics. Interaction metabolomics (Figure 7B) validated the predicted interaction between berberine and capsaicin and identified additional capsaicinoids from the *C. chinensis* extract that also appear to synergistically enhance the antimicrobial activity of berberine. On the basis of these selectivity ratio predictions, follow-up experiments could be conducted to isolate these constituents, confirm their structures with NMR, and measure the extent of their synergistic activity with berberine using checkerboard assays.

A critical element of the successful experiment with botanical extracts (Figure 7) was the chromatographic separation of the *C. chinensis* fractions. When we first set out to apply interaction metabolomics to botanical mixtures, we attempted an alternative experimental design in which we selected one partition from *H. canadensis* (HC-*aq*) and one fraction from *C. chinensis* (CCF4) and prepared mixtures from these fractions mixed at low, medium, and high concentrations (Table S11). The resulting selectivity ratio plot (Figure S17) was totally uninformative. Although some of the interaction terms for berberine and capsaicin showed up with high selectivity ratio values, many interaction terms in the data set were associated with minor constituents in the fractions that were not assignable to berberine or any of the capsaicinoids. In hindsight, this finding is not surprising. If only two fractions are used to prepare the mixtures, all components of those fractions covary in the mixtures, so all become candidates as synergists, an extreme case of confounding. Successful application of interaction metabolomics requires that both active and inactive constituents vary in their relative abundance across the mixtures employed, a requirement that can be achieved by chromatographic separation.

## Conclusions

Here we demonstrate the utility of interaction metabolomics, a new approach for predicting constituents that interact to exert a combined biological effect. In our experiments, interaction metabolomics enabled identification of synergists both in spiked fractions from a simulated extract and in combined fractions from botanical extracts. This outcome was not achievable with a classical metabolomics workflow. While the experiments presented here focused on synergistic interactions, interaction metabolomics might also prove useful to study additivity or antagonism, a possibility that could be explored in future studies.

It was critical as the first step in testing interaction metabolomics to apply an experimental design in which synergy was known to occur and in which the identities of the compounds responsible for this synergy were also known. Without this knowledge, a negative result could have been interpreted as either no synergistic interactions or a failure of the model to accurately predict synergy. Once the interaction metabolomics methodology had been developed for the mixtures of known composition, it was then possible to extend it to a more complex scenario with botanical extracts in which only some of the constituents (berberine and capsaicin) were of known identity. An obvious question that remains is whether interaction metabolomics would be effective in a scenario in which none of the active constituents of the mixture are known prior to conducting the experiment. For our studies with botanical extracts, we intentionally pursued fractions known to contain berberine and capsaicin. Had we not done so, it would have been necessary to prepare, analyze, and test a much larger number of fractions to prospect for synergists. For example, without relying on knowledge about the activity of berberine and capsaicin, we could have conducted assay-guided fractionation on the *H. canadensis* fractions and then combined the most potent antimicrobial faction with many different subfractions from *C. chinensis* at a range of concentrations. Such an experimental design would be possible, but the number of mixtures generated might necessitate a high-throughput screen with robotics for liquid handling. Finally, it is worth noting that, while we chose to validate interaction metabolomics using combinations of fractions from two different plants, it would also be possible to look for synergistic pairs in a single complex natural product extract by fractionating that extract and then recombining the fractions in various combinations and over a range of concentrations. Again, depending on the number of fractions, a high-throughput workflow might be needed for such an application.

Ultimately, our results demonstrate that, in a situation where the biological activity of a series of mixtures is due to the combined effect of two constituents, interaction metabolomics is effective for identifying the constituents that interact. The approach effectively answers the question, “Is the biological activity of these mixtures due to the synergistic interaction of multiple constituents.” As the studies with botanical extracts demonstrate, interaction metabolomics can also play a role in answering the broader and more difficult question, “Are there synergists in this extract? (Or in these two extracts)”. The challenging aspect of addressing this second question is ensuring that the synergists are present in the same fractions and at the correct combinations of concentrations to observe synergy. Satisfying these requirements requires thoughtful experimental design on the part of the analyst, relying either on some prior knowledge about the potential identities of the synergists or on a willingness and ability to prepare and test a very large number of mixtures.

The studies presented here suggest that the inclusion of interaction terms to account for possible synergistic interactions might be useful as a general approach for metabolomics data processing. In theory, it could be applicable in any scenario where two mixture components interact synergistically and would not be limited to the specific test case investigated here of an antimicrobial compound and efflux inhibitor. As such, the practice could be adopted in many fields that require identification of the chemical compounds responsible for some measurable effect, including ecology, toxicology, biomarker discovery, forensics, and many others. The broader applicability and advantages of interaction metabolomics need to be evaluated with further investigations by using additional experimental systems. Such explorations should, in theory, be quite achievable; any metabolomics experiment could be modified to account for synergistic interactions by appending the interaction terms to the data matrix. However, it is important to consider that the inclusion of interaction terms dramatically increases the number of features in the data set. Therefore, successful implementation of this approach will require careful attention to ensure that appropriate statistical methods are used, sufficient chromatographic separation has been employed to simplify the complexity of the mixtures, and feature lists have been filtered to remove noise and reduce them to a manageable size.

## Experimental Section

### General Protocol for Antimicrobial Susceptibility Assay

Antimicrobial susceptibility against *Staphylococcus aureus* (strain SA1199)^33^ was evaluated using broth microdilution methods for aerobic bacteria on the basis of the Clinical Laboratory Standards Institute (CLSI) guidelines.^34^ Cultures were grown from a single isolated colony of the strain and incubated to log-phase in a Müller-Hinton broth (MHB). The inoculum was diluted into a 96-well plate to achieve a final density of 1.0 × 10^5^ CFU/mL. Samples were introduced in triplicate and diluted in broth with a vehicle of 1% dimethyl sulfoxide (DMSO) and 1% glycerol. The negative control was vehicle alone in MHB, and the positive control was the antibiotic levofloxacin. Two sets of wells of the same samples were prepared. The first set (growth wells) contained bacterial inoculum and test compound or control in MHB. The second set of wells (control wells) had the identical composition to the growth wells except that MHB was used in place of bacterial inoculum. After 18 h of incubation, the optical density of all wells was measured at 600 nm (OD_600_) using a Synergy H1 microplate reader (Biotek, Winooski, VT, USA). The OD_600_ of each sample was corrected for the background absorbance of the sample by subtracting the measured OD_600_ of the control wells from the measured OD_600_ of the growth wells. Antimicrobial activity against *S. aureus* was calculated as percent growth inhibition relative to the vehicle control (eq 2), where vehicle OD_600_ is the background-corrected OD_600_ value for the vehicle (1% DMSO, 1% glycerol) in MHB, and sample OD_600_ is the background-corrected OD_600_ value for the treatment or control in MHB.
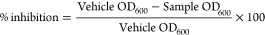
2

### Synergy Evaluation

To test the combination effects between the model compounds, a broth microdilution checkerboard assay was employed. The antimicrobial berberine (>98%, Sigma-Aldrich) and the efflux pump inhibitor piperine (>97%, Sigma-Aldrich)^1,24^ were tested in combination at concentration ranges from 4.7 to 300 μg/mL and from 0.78 to 50 μg/mL, respectively. The vehicle used was 1% DMSO and 1% glycerol in MHB. The net fractional inhibitory concentration (∑FIC) was calculated by eq 1, where MIC_berberine_ is equal to the minimum inhibitory concentration of berberine against *S. aureus* alone, MIC_piperine_ is equal to the minimum inhibitory concentration of piperine alone, MIC_berberine+piperine_ is the minimum inhibitory concentration of berberine at a given concentration of piperine, and MIC_piperine+berberine_ is equal to the minimum inhibitory concentration of piperine at a given concentration of berberine.

For the purposes of this study, combination effects were evaluated based on FIC indices, as described previously by Caesar et al., where ∑FIC ≤ 0.5 is for synergistic effects, ∑FIC between 0.5 and 1.0 is for additive effects, and ∑FIC ≥ 4.0 is for antagonistic effects.^2,35,36^

### Preparation of the Simulated Extract

A series of 42 purified natural product compounds for possible inclusion in the simulated extract was obtained from commercial sources (Table S1). The compounds included in this initial set were selected because they are known constituents of natural products and are readily available. Each compound was tested for antimicrobial activity against *Staphylococcus aureus* strain SA1199^37^ at a concentration of 256 μg/mL. Compounds that demonstrated ≥20% inhibition were rejected from the sample set. The remaining compounds were retested at 100 μM in three separate conditions, alone, in combination with 32 μg/mL berberine (95 μM), or in combination with 32 μg/mL piperine (112 μM) (Table S2). Any compound that inhibited bacterial growth by more than 20% under any of these conditions was rejected from the set to be included in the mixture.

A subset of 21 compounds from the original 42 compounds fit the selection criteria of showing ≤20% inhibition of *S. aureus* alone or in combination with berberine or piperine. The simulated extract (total mass 1288.8 mg) was prepared from these compounds as follows: naringin (8.2%), betulinic acid (0.2%), atropine (9.7%), amygdalin (12.3%), caffeine (2.9%), chlorogenic acid (2.0%), 3,4-dihydroxybenzaldehyde (2.4%), tropine (9.6%), *p*-octopamine (2.9%), boldine (10.3%), anisodamine (1.4%), quinine (7.5%), dehydroevodiamine (0.18%), apocynin (7.3%), vanillin (3.0%), ferulic acid (9.5%), vanillic acid (4.6%), syringic acid (4.9%), theobromine (0.5%), stigmasterol (0.6%), and β-sitosterol (0.2%).

### Plant Material and Extraction

The leaves of *Hydrastis canadensis* were cultivated in Hendersonville, North Carolina, in 2015, while the fruits of *C. chinense* were cultivated in Williams, Oregon, in the summer of 2022. Sample specimens of *H. canadensis* and *C. chinense* were submitted to the University of North Carolina at Chapel Hill Herbarium and were assigned accession numbers NCU583414 and NCU00445429, respectively. Finely ground dried leaves of *H. canadensis* (HC) and fruits of *C. chinense* (CC) were exhaustively extracted separately in methanol for three (3) days. The resulting extracts were dried under vacuum (HC crude, 16.0 g, and CC crude, 143 g) and then resuspended in 9:1 methanol–water followed by partitioning with hexanes to extract the fat-soluble constituents. The defatted aqueous methanolic layers of each plant extract were concentrated in vacuo to remove the methanol and then reconstituted in water followed by partitioning with chloroform. The organic layer was washed with 1% NaCl to remove the hydrosoluble tannins. The resulting aqueous and chloroform partitions of each plant extract were concentrated under vacuum followed by further drying under nitrogen yielding 7.36 g of *Hydrastis canadensis* aqueous partition (HC-*aq*) and 5.98 g of *C. chinense* chloroform partition (CC-*ch*).

### Chromatographic Separation of the Simulated Extract and Botanical Extracts

Solvents used in chromatographic separation were ACS grade (Fisher Scientific). The simulated extract and botanical extracts were dissolved completely in methanol and fractionated by normal-phase flash column chromatography on a CombiFlash RF system with a 40 g silica gel column. For the simulated extract, HC-*aq* partition, and CC-*ch* partition, gradient elution using hexane, chloroform, and methanol was employed at a flow rate of 40 mL/min for 84, 98, and 54 min, yielding 150, 196, and 87 eluates, respectively. For the simulated extract, the eluates were pooled in sets of 15 tubes to make 10 fractions. The goldenseal eluates were pooled to make three (3) fractions, and the habañero pepper eluates had 12 (12) fractions. The first pooled fraction of the simulated extract was not used because it contained an insufficient quantity of material, and the rest of the pooled simulated fractions were labeled 01–09 and were used as background matrices for preparation of the spiked fractions. The fractions of botanical extracts were labeled HCF1 to HCF3 and CCF1 to CCF12 for goldenseal and habañero pepper, respectively.

The goldenseal fraction with the highest amount of berberine (HCF2) was subjected to second-stage fractionation using reversed-phase HPLC (Varian ProStar) and injected into a Luna preparatory column (Phenomenex, 5 μm PFP, 250 mm × 21.20 mm) at a flow rate of 15 mL/min. The 30 min method started a gradient of 20% acetonitrile and 80% water with 0.1% formic acid and increased to 100% acetonitrile for 25 min. This afforded seven (7) subfractions. Meanwhile, the habañero pepper fraction with a high amount of capsaicin (CCF4) was subjected to reverse-phase HPLC and injected into a Gemini preparatory column (Phenomenex, 5 μm C18, 250 × 21.20 mm) at a flow rate of 18 mL/min. The 30 min method started with a gradient of 20:80 acetonitrile–water and increased to 100% acetonitrile for 25 min. This yielded five (5) subfractions.

### Preparation of the Botanical Mixtures

Each habañero pepper subfraction (CCF4) was spiked with the goldenseal subfraction with the highest amount of berberine (HCF2–7) to make five (5) botanical mixtures. Each mixture had a stock concentration of 5 mg/mL of CCF4-1 to CCF4-5 subfractions and 2.5 mg/mL of HCF2–7.

### Antimicrobial Evaluation of Spiked Fractions and Botanical Mixtures

Two sets of mixtures (“spiked fractions”) were prepared by spiking the inactive background matrices with berberine (M01-M08, Table 1) or with berberine and piperine (M09-M17, Table 2). Pooled fractions 01–08 were used as background matrices for M01-M08 (Table 1), and pooled fractions 01–09 were used to create M09-M17 (Table 2). Thus, pooled fractions 01–08 were used twice as background matrices, once for the berberine mixtures and once for the berberine-piperine mixtures, while pooled fraction 09 was used only once for one berberine-piperine mixture. (Fewer berberine mixtures were required for testing than berberine-piperine mixtures.) To create the mixtures with final assay concentrations shown in Tables 1 and 2, stock solutions of berberine and piperine were prepared at a concentration of 10 mg/mL in DMSO/glycerol (1:1) and combined with stock solutions of the pooled fractions. The concentration of berberine used in M01-M08 (Table 1) was slightly higher than that used in M09-M17 (Table 2) to avoid saturating the antimicrobial response due to the impact of added piperine. Three separate antimicrobial broth dilution assays were performed on the mixtures using the “General Protocol for Antimicrobial Susceptibility Testing,” one with the nine pooled fractions (background matrices) alone, one with the pooled fractions spiked with berberine, and one with the pooled fractions spiked with berberine and piperine. The botanical mixtures were tested the same way as the spiked fractions, but each mixture had an assay concentration of 100 μg/mL for CCF4-1 through CCF4-5 and 50 μg/mL for HCF2–7.

### LC-MS Analysis of Spiked Fractions and Botanical Mixtures

The spiked fractions and botanical mixtures were diluted 100-fold from assay concentration and analyzed in triplicate using a Thermo Fisher Q Exactive Plus mass spectrometer (Thermo Fisher Scientific, Waltham, MA) equipped with an electrospray ionization (ESI) source coupled with a Waters Acquity ultraperformance liquid chromatograph (Waters Corporation, Milford, MA). A 3 μL volume of each sample was injected and eluted through a reversed-phase column (BEH C18, 1.7 μm, 2.1 mm × 50 mm, Waters Corporation) using a binary solvent system consisting of water with 0.1% formic acid (solvent A) and acetonitrile with 0.1% formic acid (solvent B). The 10 min gradient elution started with 10%B for 0.5 min then increased to 100%B for 8 min and finally re-established to starting conditions in the last 1.5 min. Analysis was conducted in full scan acquisition, collecting profile data in switching positive and negative polarity. The scan range was from 120 to 1500 *m*/*z* with a scan time of 200 ms. The following mass spectrometer parameters were used: the AGC target was at 1× 10^6^ with a capillary voltage and temperature at −0.7 V and 310 °C, respectively; the S-lens RF level was 80.00, spray voltage was 3.7 kV, and the sheath and auxiliary gas flows were 50.15 and 15.16, respectively. The full MS data sets of the spiked fractions and botanical mixtures are uploaded and accessible as MassIVE data sets MSV000089598 and MSV000091287, respectively.^38,39^

### Metabolomics Data Peak Picking and Data Filtering

#### Peak Picking

The two different LC-MS data sets (M01-M08 and M09-M17) were analyzed separately. LC-MS raw files were imported into MZmine 2.53^28^ for peak picking. Methods to create a list of features include mass detection, chromatogram building, chromatogram deconvolution, deisotoping, feature alignment, gap-filling, duplicate filter, and peak filter, and the parameters used are included in Table S9. The final feature lists were imported to MS Excel for further treatment.

### Data Filtering

LC-MS data were first filtered to remove background noise, which may be due to small solvent contaminants or electronic noise in the system. The first blank filter aims to remove these signals; the relative standard deviation (RSD) of a feature across all samples, including the blanks, was calculated. All features with RSD across samples and blanks that were less than 30% (for the simulated extracts) and less than 50% (for the botanical mixtures) were removed from the data set. The next blank filter was to remove features with higher signals in the blanks as compared with the samples. This was done by calculating the percent ratio of the mean of the blanks to the mean of the samples. All features with a percent ratios higher than 80% were removed. The data sets were then filtered to remove poor quality features based on their having greater than an RSD cutoff of 35% RSD. The RSD was calculated from the feature peak areas of triplicate LC-MS analyses of the same sample. The full, RSD, and blank filtered feature lists are available as Supporting Information.^26^ Peak areas shown are the average peak areas across the three replicate injections. The second filtering step removed mass spectral features that did not vary in intensity (peak area) across the spiked fractions (mixtures), choosing an empirically selected cutoff value of ≤0.01% of the variance for the feature with highest variance for M01-M08, ≤0.1% of the variance of the features with highest variance for M09-M17, and ≤0.02% of the variance of the features with highest variance for the botanical mixtures. The reduced data set is available as Supporting Information.^26^

### Calculation of the Compound Interaction Terms

The compound interaction terms (CIT) were calculated with eq 3, where *I*_F_*i*__ represents the intensity of feature *i*, *I*_F_(*i*+1)__ represents the intensity of feature (*i* + 1), and CIT_F_*i*_F_(*i*+1)__ represents the CIT for features *i* and (*i+*1).
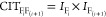
3

For a given data set, the total number of nonredundant CITs (*N*_CIT_) obtained when the features (detected ions) are combined 2 at a time is determined by eq 4, where *m* is equal to the total number of features detected across all samples.
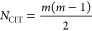
4

### Data Standardization

Highly abundant features will have a large variance that will dominate the first PLS components. Thus, they may mask other features, leading to a misinterpretation of the data. This problem is magnified when CITs are included, because the magnitude of the CITs is large compared to the magnitude of the individual features. To overcome this scaling problem, LC-MS peak areas or interaction terms were standardized to unit variance.^40^ To calculate the standardized abundance of each feature (*I*_stand, F_*i*__), the intensity of each feature (*I*_F_*i*__) in each mixture was divided by the standard deviation (*s*) of the peak area of that feature across all samples (mixtures) (eq 5).
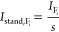
5

The standardized CIT intensities were calculated in the same fashion as the standardized feature intensities using the CIT values in place of the feature peak areas.

### Statistical Analysis: Partial-Least Squares Regression and Calculation of Selectivity Ratios

The preprocessed data sets were imported to Sirius 11.5 (Pattern Recognition Systems AS, Bergen, Norway) for statistical analysis. The data were modeled using partial least-squares regression followed by target projection (TP) to obtain selectivity ratios that connect biological activity to the mass spectral variables.^4,16,17,29^ To ensure the reliability of the model and good line fitting between the predicted and measured response variables, the root-mean-square error of prediction (RMSEP) must be calculated through validation based on repeated Monte Carlo resampling.^41−43^ The data set was validated, leaving out one object with 100 repetitions and a significance level of 0.5 to determine the number of PLS components to use for modeling. Selectivity ratios of mass spectral variables (or LC-MS features) correlating with biological activity were calculated to identify compounds of interest.^1,2,4,16,17,29,44^

## Supporting Information Available

Metabolomics Data sets of Simulated Spiked Fractions (10.5281/zenodo.7644151) LCMS Raw Data of Simulated Spiked Fractions (https://doi.org/doi:10.25345/C5NC5SH1T) LCMS Raw Data of Natural Botanical Mixtures (https://doi.org/doi:10.25345/C5H41JX33) (PDF)
