# Supplementary material for: Interaction Metabolomics to Discover Synergists in Natural Product Mixtures
Source: J Nat Prod. 2023 Apr 13;86(4):655–71. doi: 10.1021/acs.jnatprod.2c00518 (PMC10152448; doi:10.1021/acs.jnatprod.2c00518)
Supplement: Supplementary file 1 — np2c00518_si_001.pdf [file np2c00518_si_001.pdf]

Supporting Information

Interaction Metabolomics to Discover Synergists in Natural Product Mixtures

Warren S. Vidar,[a] Tim U. H. Baumeister,[b] Lindsay K. Caesar,[c] Joshua J. Kellogg,[d] Daniel A. Todd,[a] Roger G. Linington,[b] Olav M. Kvalheim,[e] and Nadja B. Cech[a]

[a]*Department of Chemistry and Biochemistry, University of North Carolina at Greensboro, Greensboro, North Carolina, 27402, United States*
[b]*Department of Chemistry, Simon Fraser University, Burnaby, BC, V5A 156, Canada*
[c]*Department of Chemistry, Northwestern University, Evanston, IL, United States*
[d]*Department of Veterinary and Biomedical Sciences, Pennsylvania State University, University Park, PA, 16802, United States*
[e]*Department of Chemistry, University of Bergen, Bergen 5020, Norway*

Table of Contents

Figure S1. Antimicrobial activity of fractions without berberine or piperine .... pg. S3

Table S1. Antimicrobial screening of 42 natural products at 256 µg/mL for *S. aureus* susceptibility testing .... pg. S4

Table S2. Antimicrobial activity of pre-selected compounds at 100 µM and in combination with berberine (32 µg/mL) and with piperine (32 µg/mL) .... pg. S5

Table S3. Natural products used in the simulated extract and analyte identification .... pg. S6

Figure S2. Antimicrobial activity of the spiked fractions, with berberine only (A) and with berberine and piperine (B) .... pg. S7

Figure S3. Positive mode full scan base peak chromatogram of M01 to M04 .... pg. S8

Figure S4. Positive mode full scan base peak chromatogram of M05 to M08 .... pg. S9

Figure S5. Positive mode full scan base peak chromatogram of M09 to M13 .... pg. S10

Figure S6. Positive mode full scan base peak chromatogram of M14 to M17 and a mixture of reference standards of compounds used in the simulated extract .... pg. S11

Figure S7. Mass spectra of berberine (A) and piperine (B) .... pg. S12

Table S4. Distribution of analytes in the spiked fractions as detected by LC-MS .... pg. S13

Table S5. Distribution of analytes in the simulated fractions after filtering features that do not vary across samples .... pg. S14

Table S6. List of feature annotations in the LC-MS data .... pg. S14-S16

Table S7. Number of features and annotated adducts after filtering .... pg. S17

Table S8. PLS modeling information for the berberine and piperine mixtures .... pg. S17

Figure S8. Comparison of the data matrices used for classical metabolomics (A) and interaction metabolomics (B) shown in Figure 4 .... pg. S18

Figure S9. Calculation of a selectivity ratio .... pg. S19

Figure S10. Positive mode base peak chromatograms of an isolated berberine standard .... pg. S20

Table S9. MZmine parameters used for peak picking analysis of the MS raw data .... pg. S21

Figure S11. Antimicrobial activity of *Hydrastis canadensis* and *Capsicum chinense* sub-fractions .... pg. S22

Figure S12. UPLC-MS chromatograms of *Hydrastis canadensis* sub-fractions .... pg. S23

Figure S13. UPLC-MS chromatograms of *Capsicum chinense* sub-fractions .... pg. S24

Figure S14. UPLC-MS chromatograms of *Capsicum chinense* and *Hydrastis canadensis* sub-fraction mixtures .... pg. S25

Figure S15. Antimicrobial activity of *Capsicum chinense* and *Hydrastis canadensis* sub-fraction mixtures .... pg. S26

Figure S16. Mass spectra of capsaicin, dihydrocapsaicin, and homodihydrocapsaicin I .... pg. S27

Table S10. PLS modeling information for the botanical extract fractions .... pg. S28

Table S11. Mixture concentrations for CCF4 combined with HC-*aq* .... pg. S29

Figure S17. Selectivity ratio plot for CCF4 combined with HC-aq .... pg. S29

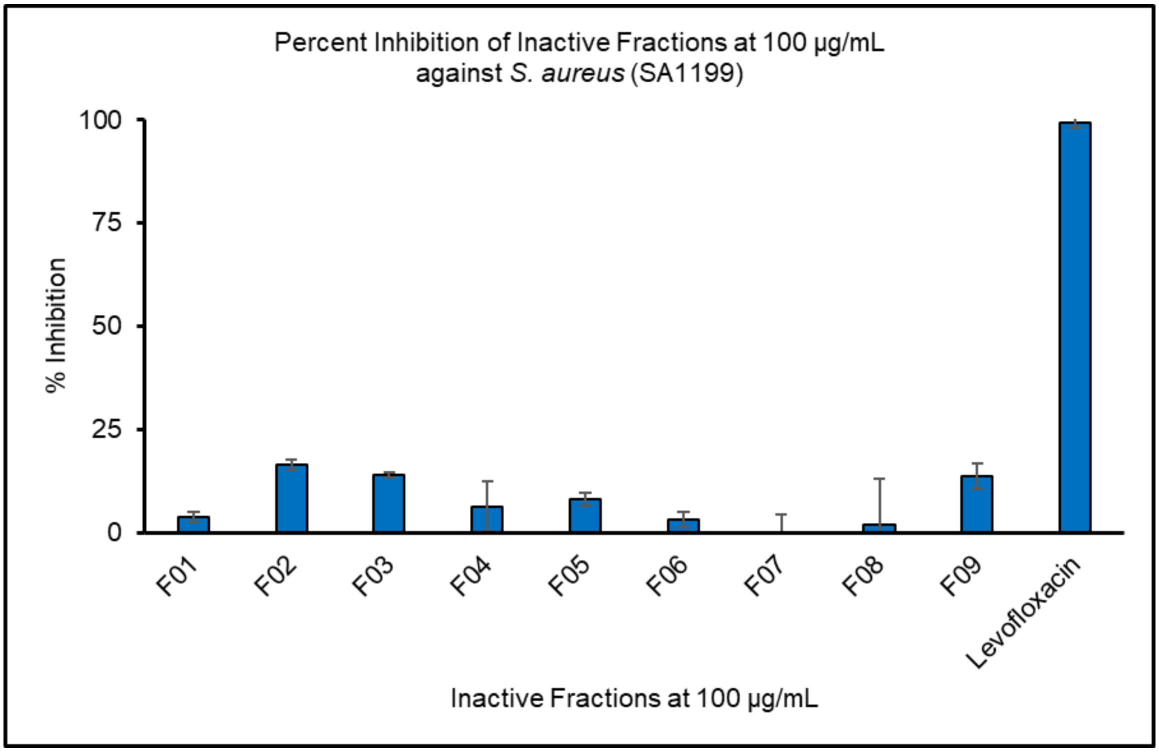

Figure S1. Antimicrobial activity against *Staphylococcus aureus* of the fractions without berberine or piperine. Error bars represent the standard deviation of triplicate % inhibition values. Percent inhibition of bacterial growth is expressed relative to the vehicle control (1% DMSO, 1% glycerol). The antibiotic levofloxacin (10 µg/mL) served as the positive control.

Table S1. Antimicrobial screening of 42 natural products at 256 μg/mL for *S. aureus* susceptibility testing

Compound Name	Percent Inhibition	Compound Name	Percent Inhibition
naringin (Sigma, >95%)	1.5 ± 4.1	p-octopamine (Cayman, >98%)	5.6 ± 4.9
beta-sitosterol (Sigma, >96%)	-9.56 ± 0.67	18β-glycyrrhetinic acid (Cayman, >98%)	57 ± 13
betulinic acid (Sigma, 90%)	8 ± 16	loganin (Cayman, >98%)	-7.1 ± 9.6
stigmasterol (Sigma, >95%)	-9.3 ± 2.1	4-hydroxycoumarin (Cayman, >98%)	84.0 ± 2.3
atropine (Sigma, >99%)	2.29 ± 0.30	7-methoxyflavone (Cayman, >98%)	20 ± 30
capsaicin (Sigma, >50%)	18.5 ± 7.1	boldine (Cayman, ≥ 95%)	0.7 ± 3.4
amygdalin (Sigma, >97%)	10.7 ± 1.9	anisodamine (Cayman, > 98%)	4.0 ± 1.6
chrysin (Sigma, >98%)	70 ± 14	harmine (Cayman, >98%)	34 ± 19
quercetin (Sigma, >98%)	94.7 ± 5.2	quinine (Cayman, >95%)	-3.4 ± 1.7
caffeine (Sigma, >99%)	3.0 ± 2.1	ursolic acid (Cayman, >98%)	21.1 ± 7.7
kaempferol (Alfa aesar, >98%)	50.9 ± 6.8	dehydroevodiamine (Cayman, >98%)	16.7 ± 1.6
myricetin (TCI, >97%)	98.41 ± 0.69	2-hydroxyanthraquinone (Cayman, >98%)	69 ± 21
chlorogenic acid (Alfa aesar, 98.2%)	16.1 ± 1.9	apocynin (Cayman, >98%)	6.1 ± 1.4
rutin (Arcos Organics, 97%)	38.2 ± 1.3	etoposide (Cayman, >98%)	99.2 ± 4.3
isorhynchophylline (Cayman, >95%)	-12.11 ± 0.74	vanillin (Alfa aesar, >99%)	-0.7 ± 3.6
3,4-dihydroxybenzaldehyde (Cayman, >98%)	11.3 ± 4.8	coumarin (TCI, >99%)	67.8 ± 3.0
palmatine (Cayman, >98%)	99.04 ± 0.07	salicylic acid (SCBT, 99%)	41.1 ± 3.6
tropine (Cayman, >95%)	-0.1 ± 2.3	ferulic acid (Cayman, >98%)	8.0 ± 2.6
chrysosplenetin (Cayman, >98%)	-12.5 ± 1.9	vanillic acid (SCBT, 97%)	20.3 ± 3.5
naringenin (Cayman, >98%)	94.7 ± 4.0	syringic acid (Cayman, 98%)	8.9 ± 1.5
berberine (Sigma, >98%)	99.84 ± 0.12	theobromine (Cayman, >98%)	5.9 ± 4.2

Table S2. Antimicrobial activity of pre-selected compounds at 100 µM and in combination with berberine (32 µg/mL) and with piperine (32 µg/mL)

Compound Name	Percent Inhibition at 100 µM[a]		
	Compound only	With berberine at 32 µg/mL	With piperine at 32 µg/mL
naringin	3.0 ± 1.4	35.1 ± 1.6	13.1 ± 1.6
betulinic acid	13 ± 14	27 ± 13	11 ± 14
atropine	4.48 ± 0.70	41.93 ± 0.77	18.2 ± 5.1
capsaicin	-16.8 ± 2.0	99.16 ± 0.13	16.4 ± 1.7
amygdalin	1.0 ± 2.9	32.4 ± 3.1	15.29 ± 0.95
caffeine	4.8 ± 1.6	35.29 ± 0.57	17.5 ± 1.3
chlorogenic acid	6.46 ± 0.78	36.1 ± 1.8	20.8 ± 4.2
3,4-dihydroxybenzaldehyde	2.3 ± 2.1	29.1 ± 3.2	18.2 ± 4.3
tropine	0.4 ± 3.1	31.6 ± 3.9	14.5 ± 2.4
p-octopamine	4.3 ± 1.1	35.10 ± 0.35	18.1 ± 1.1
boldine	4.5 ± 1.6	34.3 ± 2.6	16.6 ± 1.1
anisodamine	0.8 ± 3.4	29.7 ± 3.2	15.1 ± 2.9
quinine	-1.6 ± 3.7	29.1 ± 1.6	11.2 ± 2.8
dehydroevodiamine	6.3 ± 1.5	32.0 ± 2.4	19.1 ± 1.1
apocynin	5.4 ± 1.8	28.31 ± 0.92	16.5 ± 1.4
vanillin	2.5 ± 2.6	24.6 ± 6.5	17.1 ± 1.8
ferulic acid	-0.9 ± 7.3	29.2 ± 2.9	16.5 ± 3.0
vanillic acid	5.4 ± 1.2	32.7 ± 1.8	14.3 ± 1.8
syringic acid	4.38 ± 0.65	29.84 ± 0.59	15.7 ± 2.0
theobromine	0.3 ± 4.0	25.8 ± 6.6	15.4 ± 2.7
berberine	2.0 ± 7.2	48.6 ± 1.9	99.35 ± 0.11
piperine	-0.9 ± 1.6	99.12 ± 0.18	23.1 ± 3.2

a. Values are reported as mean % inhibition of biological replicates (triplicate wells) ± standard deviation.

Table S3. List of natural products used in the simulated extract and analyte identification

List of Compounds in the Simulation Mixture			Experimentally Measured Accurate Mass and Retention Time[a]					
Compound Name	Molar Mass	Mono-isotopic Mass	$[M+H]^+$	POS	RT	$[M-H]^-$	NEG	RT
naringin	580.54	580.1792	581.1870	581.1860	2.59	579.1714	579.1719	2.59
betulinic acid	456.70	456.3604	457.3682	457.3661	7.51	455.3526	455.3528	7.49
atropine	289.37	289.1678	290.1756	290.1747	2.06	288.1600	n.d.	n.d.
amygdalin	457.43	457.1584	458.1662	458.1650	1.65	456.1506	456.1505	1.65
caffeine	194.19	194.0804	195.0882	195.0875	1.49	193.0726	n.d.	n.d.
chlorogenic acid	354.31	354.0951	355.1029	355.1018	1.37	353.0873	353.0877	1.37
3,4-dihydroxybenzaldehyde	138.12	138.0317	139.0395	139.0389	1.44	137.0239	n.d.	n.d.
tropine	141.21	141.1154	142.1232	142.1226	0.51	140.1076	n.d.	n.d.
p-octopamine	189.64	153.0790	154.0868	154.0862	0.51	152.0712	n.d.	n.d.
boldine	327.38	327.1471	328.1549	328.1538	1.81	326.1393	326.1404	1.84
anisodamine	305.37	305.1627	306.1705	306.1695	1.49	304.1549	n.d.	n.d.
quinine	324.42	324.1838	325.1916	325.1905	1.93	323.1760	n.d.	n.d.
dehydroevodiamine	337.80	301.1215	302.1293	302.1285	2.93	300.1137	n.d.	n.d.
apocynin	166.17	166.0630	167.0708	167.0703	2.40	165.0552	165.1546	2.40
vanillin	152.15	152.0473	153.0551	153.0546	2.20	151.0395	151.0389	2.19
ferulic acid	194.18	194.0579	195.0657	195.0651	2.37	193.0501	193.0498	2.36
vanillic acid	168.15	168.0423	169.0501	169.0494	1.72	167.0345	167.0336	1.73
syringic acid	198.17	198.0528	199.0606	199.0599	1.80	197.0450	197.0447	1.81
theobromine	180.16	180.0647	181.0725	181.0718	0.78	179.0569	n.d.	n.d.
β-sitosterol	414.71	415.3934	n.d.	n.d.	n.d.	n.d.	n.d.	n.d.
stigmasterol	412.69	413.3777	n.d.	n.d.	n.d.	n.d.	n.d.	n.d.
berberine	371.81	336.1236	336.1236	336.1226	3.19	335.1158	n.d.	n.d.
piperine	285.34	285.1365	286.1443	286.1434	4.96	284.1287	n.d.	n.d.

[a]n.d. = not detected

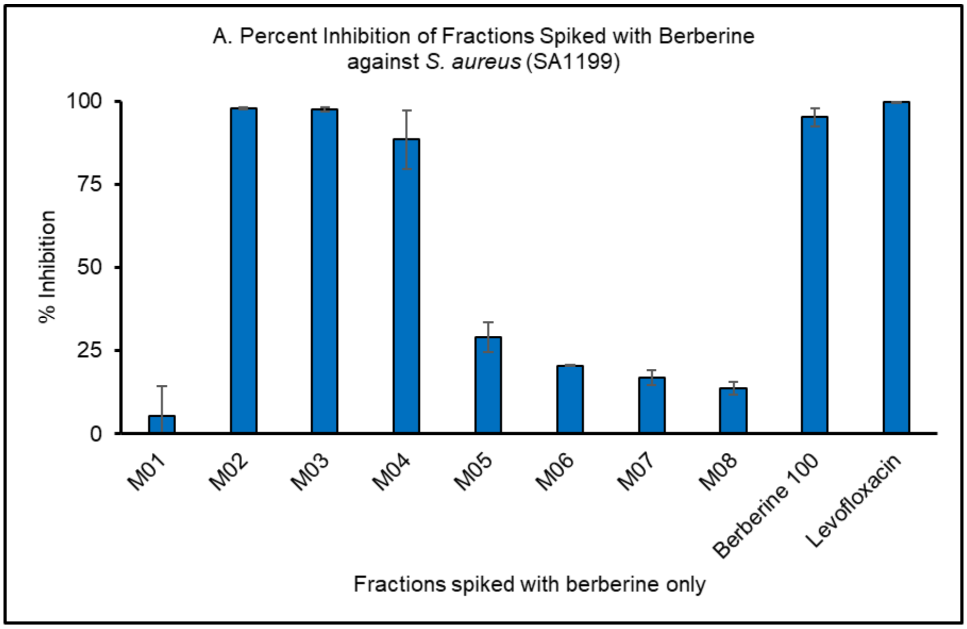

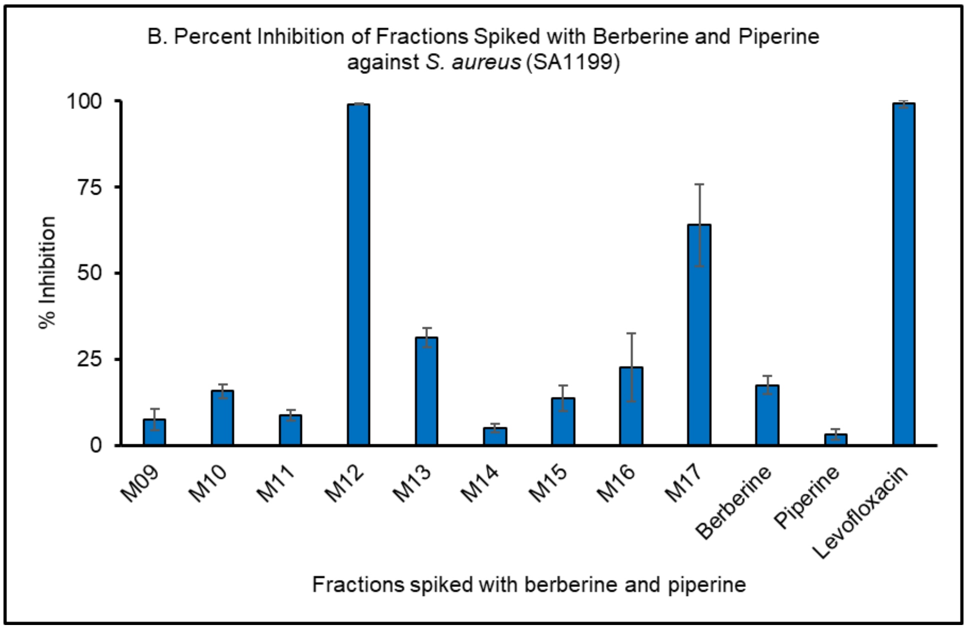

Figure S2. Antimicrobial activity against *Staphylococcus aureus* of the spiked fractions, with berberine only (A) and with berberine and piperine (B). Error bars represent the standard deviation of triplicate % inhibition values. Percent inhibition of bacterial growth is expressed relative to the vehicle control (1% DMSO, 1% glycerol). Levofloxacin (10 µg/mL) served as the positive control as a known antibiotic. In A, berberine at 100 µg/mL was also added as a control. The synergistic enhancement of biological activity by piperine is apparent in **Figure S2B**. For example, mixture ten (M10) contains 32 µg/mL berberine and only exhibits 15.8 ± 2.0% inhibition of *S. aureus* growth. Mixture twelve (M12) contains the same amount of berberine as M10 (32 µg/mL) but also contains 32 µg/mL piperine and demonstrates 99.12 ± 0.15% inhibition. Since piperine demonstrates no antimicrobial activity alone, the enhanced biological activity of M12 as compared to M10 represents synergy. A similar conclusion can be drawn by comparing the biological activity of M15 to M17 **(Figure S2B)**.

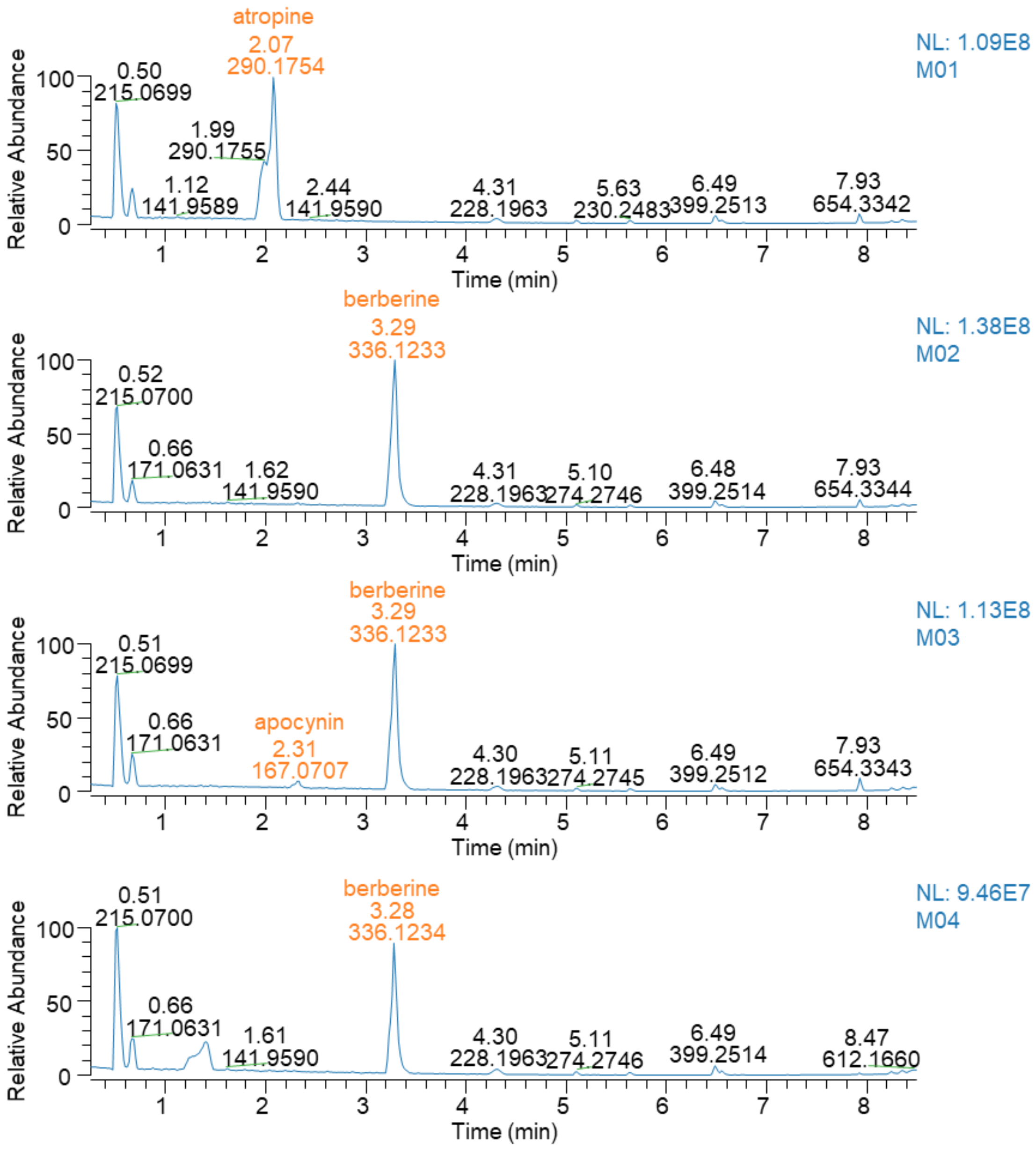

Figure S3. Positive mode full scan base peak chromatogram of M01 to M04. Not all analytes of interest are evident in the base peak chromatogram, but many could be identified with selected ion chromatograms, as indicated in Tables S7 and S8.

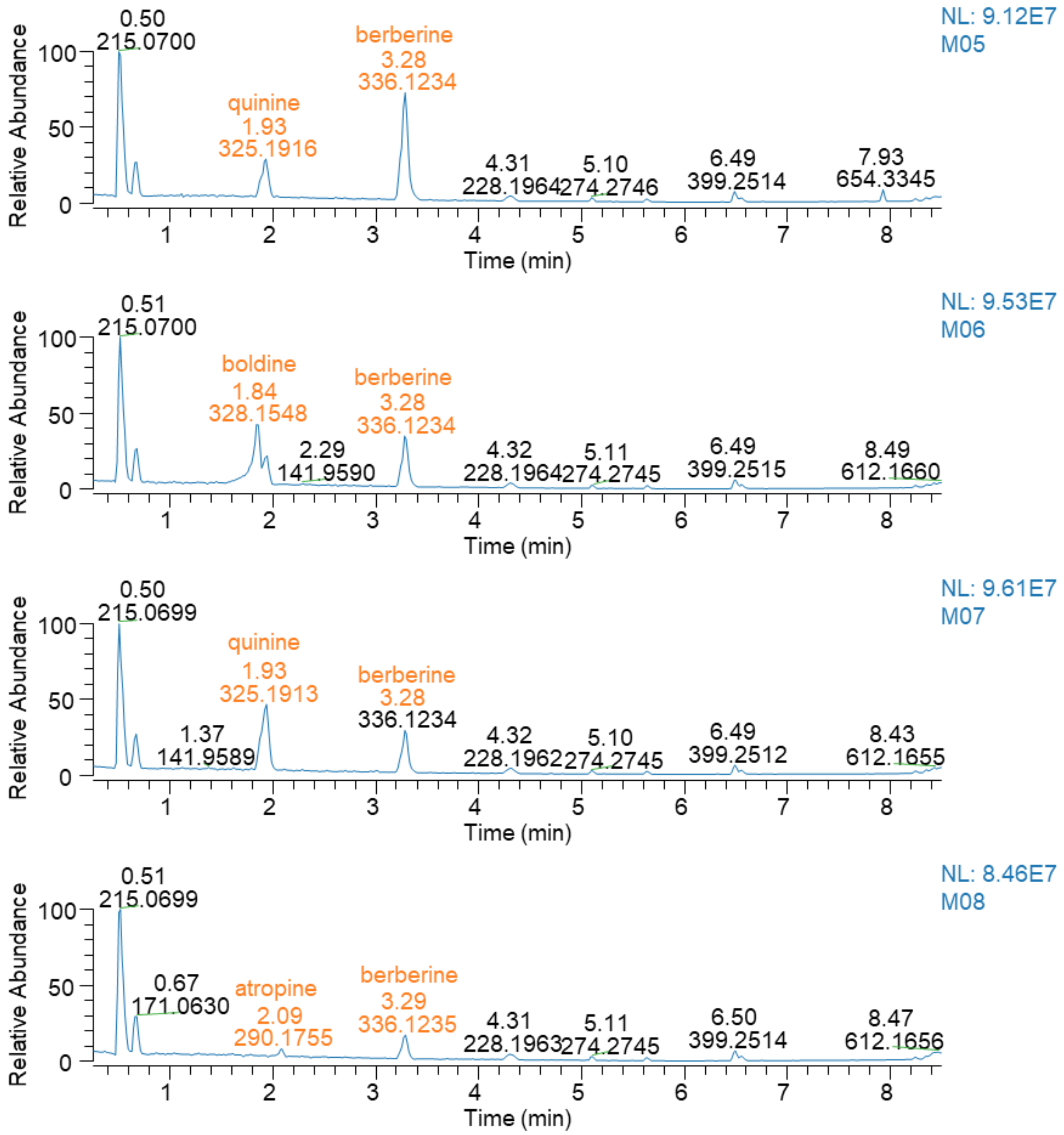

Figure S4. Positive mode full scan base peak chromatogram of M05 to M08. Not all analytes of interest are evident in the base peak chromatogram, but many could be identified with selected ion chromatograms, as indicated in Tables S7 and S8.

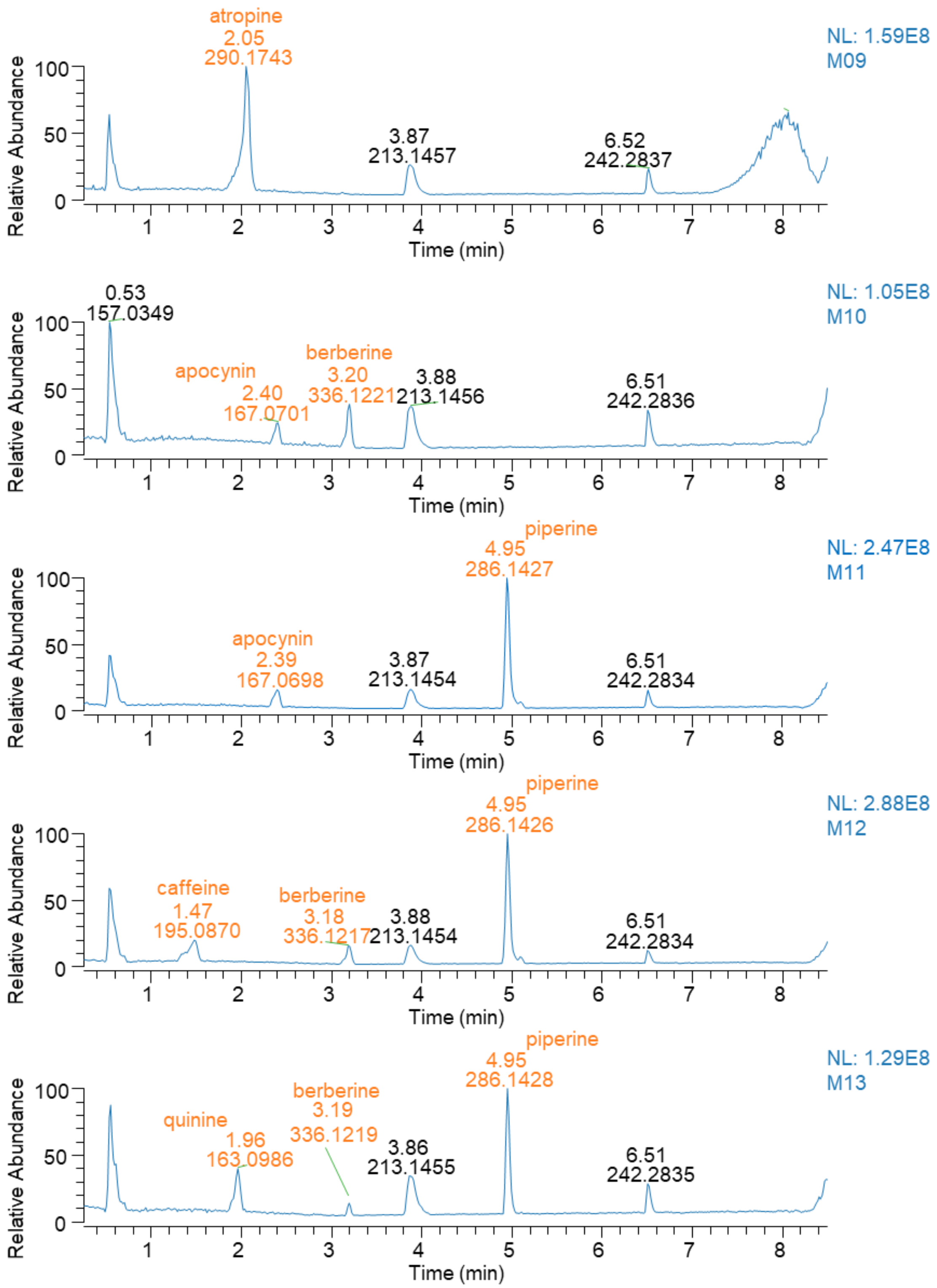

Figure S5. Positive mode full scan base peak chromatogram of M09 to M13. Not all analytes of interest are evident in the base peak chromatogram, but many could be identified with selected ion chromatograms, as indicated in Tables S7 and S8.

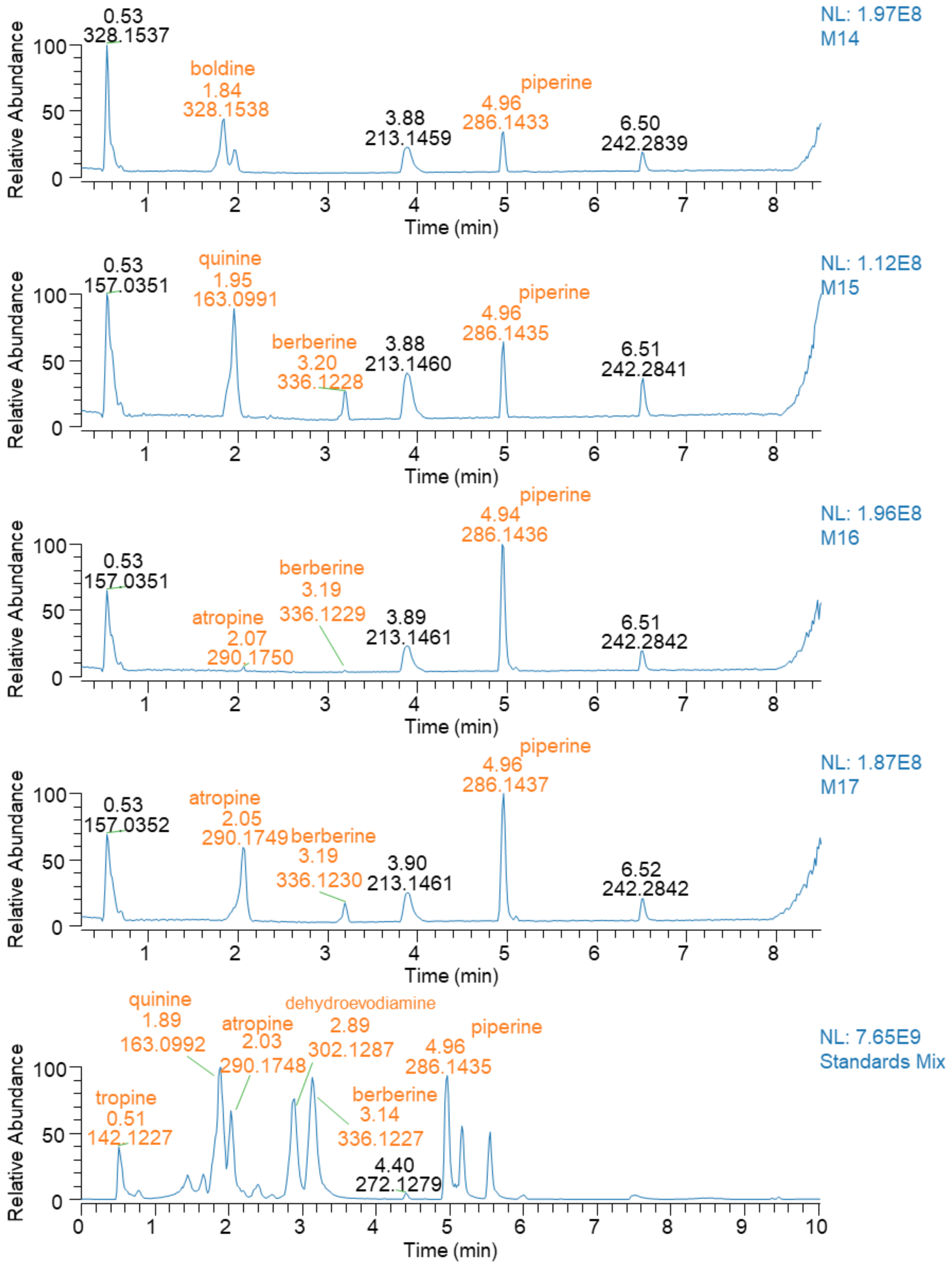

Figure S6. Positive mode full scan base peak chromatogram of M14 to M17 and a mixture of reference standards of compounds used in the simulated extract. Not all analytes of interest are evident in the base peak chromatogram, but many could be identified with selected ion chromatograms, as indicated in Tables S7 and S8.

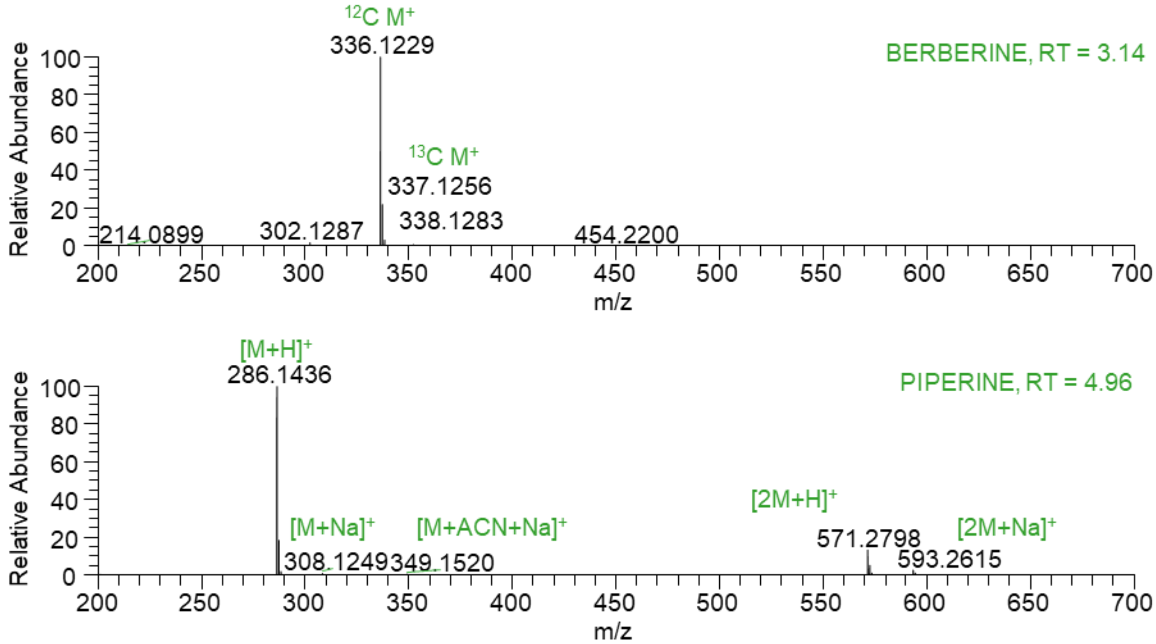

Figure S7. Mass spectra of berberine (A) and piperine (B). Figure A shows the ^{13}C isotope of berberine, while Figure B highlights multiple peaks corresponding to different adducts of piperine. RT refers to the retention time of the peak (time eluted from the LC column) in minutes.

Table S1. Distribution of analytes in the spiked fractions as detected by LC-MS after filtering based on <35% relative standard deviation in peak area across replicate injections.

Analyte	M01	M02	M03	M04	M05	M06	M07	M08	M09	M10	M11	M12	M13	M14	M15	M16	M17
naringin								■								■	
betulinic acid											■	■					
atropine	■						■	■	■						■	■	■
amygdalin								■								■	■
caffeine				■	■							■	■				
chlorogenic acid									■							■	■
3,4-dihydroxybenzaldehyde	■				■	■	■		■				■	■	■	■	■
tropine	■								■								■
p-octopamine																	■
boldine	■					■	■		■					■	■	■	■
anisodamine	■								■								■
quinine	■				■	■	■	■	■				■	■	■	■	■
dehydroevodiamine	■						■	■	■						■		■
apocynin		■	■	■						■	■	■	■				
vanillin		■	■							■	■	■	■	■			
ferulic acid					■	■	■							■	■	■	
vanillic acid														■	■	■	
syringic acid					■									■	■	■	
theobromine													■				
β-sitosterol																	
stigmasterol																	
berberine		■	■	■	■	■	■	■		■		■	■	■	■	■	■
piperine											■	■	■	■	■	■	■

Grey– analyte identified
White – not detected

Table S2. Distribution of analytes in the simulated fractions after filtering features that do not vary across samples

Analyte	M01	M02	M03	M04	M05	M06	M07	M08	M09	M10	M11	M12	M13	M14	M15	M16	M17
naringin								■									
betulinic acid																	
atropine	■						■	■	■						■	■	■
amygdalin								■								■	■
caffeine				■	■							■	■				
chlorogenic acid																	
3,4-dihydroxybenzaldehyde	■				■	■	■		■				■	■	■	■	■
tropine																	
p-octopamine																	
boldine	■					■	■		■					■	■	■	■
anisodamine	■								■								■
quinine	■				■	■	■	■	■				■	■	■	■	■
dehydroevodiamine	■						■	■	■								
apocynin		■	■	■						■	■	■	■				
vanillin										■	■	■	■	■			
ferulic acid					■	■	■							■	■	■	
vanillic acid																	
syringic acid					■												
theobromine																	
β-sitosterol																	
stigmasterol																	
berberine		■	■	■	■	■	■	■		■		■	■	■	■	■	■
piperine											■	■	■	■	■	■	■

[a]Grey – analyte identified
[b]White – not detected or filtered out
[c]% Variance cut-off: M01-M08 = 0.1%; M09-M14 = 0.01%

Table S3. List of feature annotations in the LC-MS data

Features (*m/z* -TR)[a]	Molecular Ion or Adduct[b]	Compound Name (Analyte)
137.0232-1.43	$M\text{-}H]^-$	3,4-dihydroxybenzaldehyde
139.0389-1.44	$[M+H]^+$	3,4-dihydroxybenzaldehyde
142.1226-0.52	$[M+H]^+$	tropine
151.0388-2.2	$[M\text{-}H]^-$	vanillin
153.0544-2.19	$[M+H]^+$	vanillin
154.0862-0.53	$[M+H]^+$	p-octopamine
163.0991-1.96	$[M+2H]^{2+}$	quinine
165.0545-2.4	$[M\text{-}H]^-$	apocynin
167.0339-1.73	$[M\text{-}H]^-$	vanillic acid
167.07-2.4	$[M+H]^+$	apocynin
169.0497-1.72	$[M+H]^+$	vanillic acid
173-1.41	$[M+Cl]^-$	3,4-dihydroxybenzaldehyde
176.0681-0.51	$[M+Na]^+$	p-octopamine
181.0718-0.78	$[M+H]^+$	theobromine
183.6123-1.94	$[M+ACN+2H]^{2+}$	quinine
193.0498-2.36	$[M\text{-}H]^-$	ferulic acid
194.0807-2.2	$[M+ACN+H]^+$	vanillin
195.0651-2.36	$[M+H]^+$	ferulic acid
195.0873-1.48	$[M+H]^+$	caffeine
197.0447-1.79	$[M\text{-}H]^-$	syringic acid
199.0599-1.79	$[M+H]^+$	syringic acid
203.0537-0.78	$[M+Na]^+$	theobromine
222.0983-0.78	$[M+ACN+H]^+$	theobromine
229.0268-2.35	$[M+Cl]^-$	ferulic acid
236.0913-2.37	$[M+ACN+H]^+$	ferulic acid
244.0801-0.79	$[M+ACN+Na]^+$	theobromine
251.017-1.44	$[M+TFA\text{-}H]^-$	3,4-dihydroxybenzaldehyde
275.0562-1.44	$[2M\text{-}H]^-$	3,4-dihydroxybenzaldehyde
286.1433-4.95	$[M+H]^+$	piperine
290.1746-2.06	$[M+H]^+$	atropine
302.1285-2.94	$[M+H]^+$	dehydroevodiamine
304.1919-5.16	$[M\text{-}H]^-$	capsaicin
306.1696-1.48	$[M+H]^+$	anisodamine
306.2062-5.16	$[M+H]^+$	capsaicin
308.125-4.95	$[M+Na]^+$	piperine
312.1558-2.06	$[M+Na]^+$	atropine
324.0991-4.95	$[M+K]^+$	piperine
325.1905-1.96	$[M+H]^+$	quinine
328.1539-1.83	$[M+H]^+$	boldine

328.1878-5.16	$[M+Na]^+$	capsaicin
334.1811-0.54	$[2M+3H_2O+2H]^{2+}$	p-octopamine
336.1224-3.19	$[M+H]^+$	berberine
340.1319-1.47	$[M+Cl]^-$	anisodamine
340.1686-5.16	$[M+Cl]^-$	capsaicin
344.1619-5.16	$[M+K]^+$	capsaicin
349.1516-4.96	$[M+ACN+Na]^+$	piperine
350.1612-1.47	$[M+FA-H]^-$	anisodamine
350.1975-5.16	$[M+FA-H]^-$	capsaicin
353.0879-1.36	$[M-H]^-$	chlorogenic acid
355.1019-1.37	$[M+H]^+$	chlorogenic acid
359.1532-1.94	$[M+Cl]^-$	quinine
361.1361-0.78	$[2M+H]^+$	theobromine
369.182-1.93	$[M+FA-H]^-$	quinine
372.1286-1.36	$[M+NH_4]^+$	chlorogenic acid
375.0689-1.36	$[M+Na-2H]^-$	chlorogenic acid
377.0837-1.37	$[M+Na]^+$	chlorogenic acid
383.1182-0.78	$[2M+Na]^+$	theobromine
389.0646-1.36	$[M+Cl]^-$	chlorogenic acid
393.0577-1.35	$[M+K]^+$	chlorogenic acid
399.0931-1.36	$[M+FA-H]^-$	chlorogenic acid
455.3528-7.5	$[M-H]^-$	betulinic acid
456.1505-1.65	$[M-H]^-$	amygdalin
458.1649-1.65	$[M+H]^+$	amygdalin
475.1919-1.66	$[M+NH_4]^+$	amygdalin
480.1472-1.65	$[M+Na]^+$	amygdalin
492.1282-1.66	$[M+Cl]^-$	amygdalin
496.1207-1.65	$[M+K]^+$	amygdalin
498.394-7.5	$[M+ACN+H]^+$	betulinic acid
501.3585-7.5	$[M+FA-H]^-$	betulinic acid
502.1292-1.65	$[M+2Na-H]^+$	amygdalin
502.1569-1.65	$[M+FA-H]^-$	amygdalin
570.1443-1.65	$[M+TFA-H]^-$	amygdalin
571.2795-4.95	$[2M+H]^+$	piperine
579.1724-2.59	$[M-H]^-$	naringin
579.3422-2.06	$[2M+H]^+$	atropine
581.1863-2.59	$[M+H]^+$	naringin
593.2612-4.95	$[2M+Na]^+$	piperine
598.2128-2.59	$[M+NH_4]^+$	naringin
603.1681-2.58	$[M+Na]^+$	naringin
603.2496-2.93	$[2M+H]^+$	dehydroevodiamine
609.2343-4.95	$[2M+K]^+$	piperine
611.4051-5.16	$[2M+H]^+$	capsaicin

615.1492-2.59	$[M+Cl]^-$	naringin
625.1781-2.58	$[M+FA-H]^-$	naringin
633.387-5.15	$[2M+Na]^+$	capsaicin
649.3612-5.16	$[2M+K]^+$	capsaicin
649.3739-1.92	$[2M+H]^+$	quinine
693.1652-2.59	$[M+TFA-H]^-$	naringin
707.1834-1.36	$[2M-H]^-$	chlorogenic acid
731.1777-1.36	$[2M+Na]^+$	chlorogenic acid
911.7138-7.5	$[2M-H]^-$	betulinic acid
913.3073-1.66	$[2M-H]^-$	amygdalin
913.3111-1.64	$[2M-H]^-$	amygdalin
937.3046-1.64	$[2M+Na]^+$	amygdalin
937.3082-1.65	$[2M+Na]^+$	amygdalin
959.3157-1.65	$[2M+FA-H]^-$	amygdalin
1159.3512-2.59	$[2M-H]^-$	naringin
1161.364-2.59	$[2M+H]^+$	naringin
1161.3663-2.59	$[2M+H]^+$	naringin
1205.3577-2.58	$2M+FA-H]^-$	naringin

[a] Each feature is described by its measured mass to charge ratio (*m/z*) and retention time (TR, min.)
[b]ACN = acetonitrile, FA = formic acid, TFA = trifluoroacetic acid,.

Table S4. Number of features and annotated adducts after the different filtering steps

**Data Filtering for M01 to M08**			**Data Filtering for M09 to M17**		
Data Filtering	No. of Features	No. of Annotated Adducts	Data Filtering	No. of Features	No. of Annotated Adducts
Exported from MZmine	2894	38	Exported from MZmine	5698	52
After Blank Filter	446	32	After Blank Filter	1696	51
After RSD Filter	232	30	After RSD Filter	817	50
After variance across samples filter	26	15	After variance across samples filter	33	18

Table S5. PLS Modeling Information for the piperine and berberine spiked mixtures

	**M09-M17 (Fractions spiked with berberine and piperine)**		**M01-M08 (Fractions spiked with berberine)**	
	**Classical Model Fig. 5B**	**Interaction Model Fig. 5C**	**Classical Model Fig. 5A**	**Interaction Model Fig. 5D**
Object count	9	9	8	8
Variable count	33	462	26	207
Comp. retained	2	3	2	3
Comp. variance (x, y)	1 - 30.50%, 76.47;<br>2 - 22.27%, 14.26%	1 – 22.33%, 75.74%<br>2 – 20.44%, 16.25%<br>3 – 20.38%, 6.48%	1 – 24.42%, 95.89%<br>2 – 15.00%, 3.18%	1 – 19.61%, 86.37%<br>2 – 21.79%, 12.27%<br>3 – 23.06%, 1.03%
Monte Carlo validation threshold	0.357	0.333	0.500	0.500
RMSEP	14.02	11.09	18.61	27.22
R^2Y	0.907	0.985	0.991	0.997

A. Classical Metabolomics Matrix

Sample	Activity	F1	F2	F3	...	Fm
M1	A_{M1}	$I_{F1,M1}$	$I_{F2,M1}$	$I_{F3,M1}$	...	$I_{Fm,M1}$
M2	A_{M2}	$I_{F1,M2}$	$I_{F2,M2}$	$I_{F3,M2}$	...	$I_{Fm,M2}$
M3	A_{M3}	$I_{F1,M3}$	$I_{F2,M3}$	$I_{F3,M3}$	...	$I_{Fm,M3}$
⋮	⋮	⋮	⋮	⋮		⋮
Mn	A_{Mn}	$I_{F1,Mn}$	$I_{F2,Mn}$	$I_{F3,Mn}$		$I_{Fm,Mn}$

B. Interaction Metabolomics Matrix

							Compound Interaction Terms (CIT)		
Sample	Activity	F1	F2	F3	...	Fm	F1 × F2	F1 × F3	...
M1	A_{M1}	$I_{F1,M1}$	$I_{F2,M1}$	$I_{F3,M1}$	...	$I_{Fm,M1}$	$I_{F1,M1} \times I_{F2,M1}$	$I_{F1,M1} \times I_{F3,M1}$	...
M2	A_{M2}	$I_{F1,M2}$	$I_{F2,M2}$	$I_{F3,M2}$	...	$I_{Fm,M2}$	$I_{F1,M2} \times I_{F2,M2}$	$I_{F1,M2} \times I_{F3,M2}$	...
M3	A_{M3}	$I_{F1,M3}$	$I_{F2,M3}$	$I_{F3,M3}$	...	$I_{Fm,M3}$	$I_{F1,M3} \times I_{F2,M3}$	$I_{F1,M3} \times I_{F3,M3}$	...
⋮	⋮	⋮	⋮	⋮		⋮	⋮	⋮	⋮
Mn	A_{Mn}	$I_{F1,Mn}$	$I_{F2,Mn}$	$I_{F3,Mn}$		$I_{Fm,Mn}$	$I_{F1,Mn} \times I_{F2,Mn}$	$I_{F1,Mn} \times I_{F3,Mn}$	...

Figure S8. Comparison of the data matrices used for classical metabolomics (A) and interaction metabolomics (B) shown in **Figure 4**. Each matrix contains data for all mixtures (***M***) in which features (***F***) are detected. A feature (***F***) represents a peak in the LC-MS dataset with a unique *m/z* value and retention time. The intensity (***I***) of each feature in each mixture is obtained by integrating the relevant selected ion trace in the LC-MS chromatogram. The intensities (***I***) of the features vary between mixtures because the abundance of the compounds (analytes) associated with the features differs between the mixtures. The data matrix used for the interaction workflow (B) includes the same features described for the classical metabolomics matrix (A), but also includes additional compound interaction terms (CITs) **(Equation 3)** for each pair of features detected. The total number of mixtures is ***n*** and the total number of features is m. Each mixture has a measured biological activity ($\boldsymbol{A_{Mn}}$), which in this study is measured % inhibition against *Staphylococcus aureus*. CITs are included in matrix B (for interaction metabolomics) and are obtained by multiplying together the intensities (***I***) of the features in a pairwise fashion **(Equation 3)**. For classical metabolomics (A), the first column on the matrix contains biological activity ($\boldsymbol{A_{Mn}}$) for each mixture ($\boldsymbol{M_n}$). For each mixture, there are a series of many additional columns containing the intensity ($\boldsymbol{I_{Fn,Mn}}$) values for each feature ($\boldsymbol{F_n}$) in each mixture ($\boldsymbol{M_n}$). Prior to final data analysis, these data matrices are normalized to unit variance as shown by **Equation 5**.

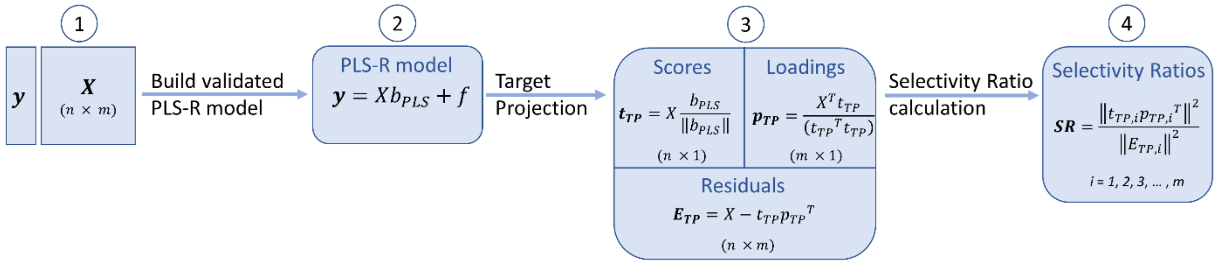

Figure S9. Calculation of selectivity ratios. (1) The data matrix has dimensions of n rows and m columns. It consists of a response vector $\boldsymbol{y}$ (biological dataset) with (indicated as $\boldsymbol{A_{Mn}}$ in **Figure S8**) and a corresponding $\boldsymbol{X}$ predictor matrix. In the case of the conventional workflow, the $\boldsymbol{X}$ predictor matrix is composed of a series of intensities ($\boldsymbol{I}$) for all detected features **(Figure S8A)**. In the case of the synergy workflow, the $\boldsymbol{X}$ predictor matrix also includes the compound interaction terms **(Figure S8B)**. (2) The regression coefficients $\boldsymbol{b}_{\mathrm{PLS}}$ from the PLS-R model are used to perform the Target Projection (TP). (3) The TP splits the dataset into predictive loadings $\boldsymbol{p}_{\mathrm{TP}}$ and scores $\boldsymbol{t}_{\mathrm{TP}}$ and a residual matrix $\boldsymbol{E}_{\mathrm{TP}}$ (dimensions in parentheses). (4) For each feature, a selectivity ratio (SR) is calculated.

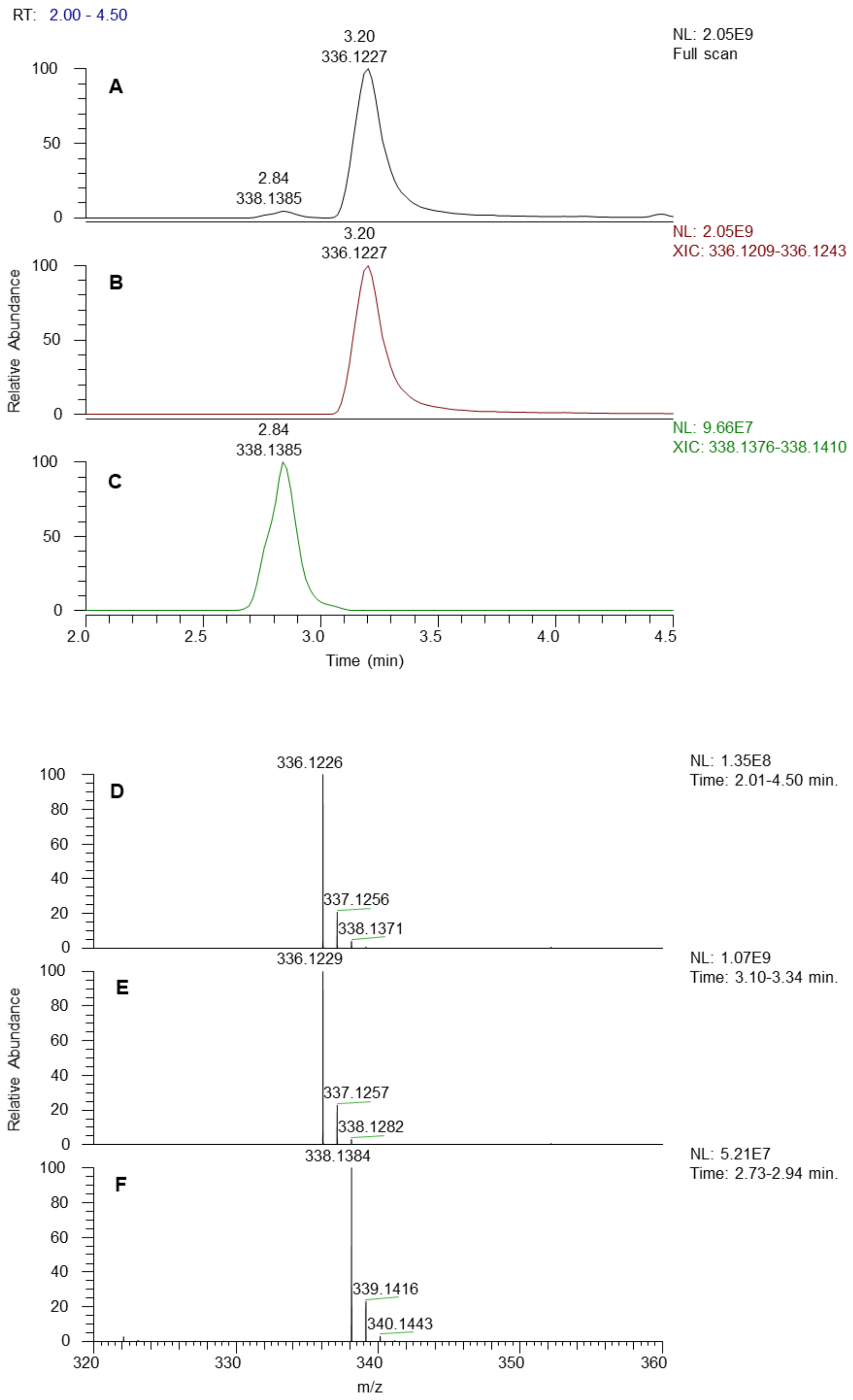

Figure S10. Positive mode base peak chromatograms of an isolated berberine standard. Figure A shows the full scan within the time range of 2.00 to 4.50 min. Figure B shows the selected ion chromatogram (XIC) for berberine at *m/z* 336.1227, while figure C shows the selected ion chromatogram (XIC) for a possible impurity, which is putatively identified as 7,8-dihydroberberine at *m/z* 338.1385. The two ions have different retention time, making the latter not an adduct or isotope of berberine. Figure D to F show the mass spectra of the full scan and selected ion chromatograms of berberine and putative 7,8-dihydroberberine. NL represents the “normalization level” or the intensity valued detected by the mass spectrometer used to normalize the abundance on the y-axis.

Table S6. MZmine parameters used for peak picking analysis of the MS raw data

**LCMS File No.: 210723 and 220202**			**By: Warren Vidar**	
**Parameters**	**Recommendations**	**210723 (M09-M17)**	**220202 (M01-M08)**	**230128 (Botanical Mix)**
**Raw data methods > Filtering > Crop Filter (Optional)**				
**RT**	0.00 to wash time	0.00 to 8.00	0.00 to 8.00	0.00 to 8.00
**Polarity**	POS / NEG	POS & NEG	POS & NEG	POS & NEG
**Spectrum type**	Profile / Centroid	Profile	Profile	Centroid
m/z	Auto range	Auto range	Auto range	Auto range
**Raw data methods > Feature detection > Mass Detection**				
**Mass detector**	Exact mass / Centroid	Exact mass	Exact mass	Centroid
**MS1 Noise level**	5E3 to 5E4	5E3	5E3	5E3
**MS2 Noise level**	0	0	0	5E2
**Raw data methods > Feature detection > ADAP Chromatogram Builder**				
**No. of scans**	4 or 5	4	5	5
**Group intensity threshold**	5E3	5E3	5E3	5E3
**Min highest intensity**	1E5 to 5E5	1E5	1E5	1E5
***m/z* tolerance**	0.003 Da	0.003 Da	0.003 Da	0.003 Da
**Feature list methods > Feature detection > Chromatogram Deconvolution**				
**Algorithm**	Wavelets ADAP	Wavelets ADAP	Wavelets ADAP	Wavelets ADAP
***m/z* center calculation**	Median	Median	Median	Median
***m/z* range for MS2 scan pairing (Da)**	0.025 Da	n/a	n/a	0.025
**RT range for MS2 scan pairing (min.)**	0.08 min.	n/a	n/a	0.08
**S/N threshold**	10	10	10	10
**S/N estimator**	Intensity window SN	Intensity window SN	Intensity window SN	Intensity window SN
**Min feature height**	1E5	1E5	1E5	1E5
**Coefficient/area threshold**	30 to 200	50	40	50
**Peak duration range**	0.00 to 2.00	0.00 to 2.00	0.00 to 2.00	0.00 to 2.00
**RT wavelet range**	0.00 to 0.10	0.00 to 0.10	0.00 to 0.10	0.00 to 0.10
**Isotopes > Isotopic Peaks Grouper**				
***m/z* tolerance**	0.0015 Da	0.0015 Da	0.0015 Da	0.0015 Da
**RT tolerance**	0.05 min.	0.05 min.	0.05 min.	0.05 min.
**Maximum charge**	3	3	3	3
**Representative isotope**	Most intense	Most intense	Most intense	Most intense
**Feature list methods > Alignment > Join Aligner**				
***m/z* tolerance**	0.0015 Da	0.0015 Da	0.0015 Da	0.0015 Da
**Weight for m/z**	2	2	2	2
**RT tolerance**	0.05 min.	0.05 min.	0.05 min.	0.05 min.
**Weight for RT**	1	1	1	1
**Require same charge state**	checked	checked	checked	checked
**Compare isotope pattern**	checked	checked	checked	checked
**Isotope m/z tolerance**	0.0015 Da	0.0015 Da	0.0015 Da	0.0015 Da
**Min. absolute intensity**	1E5 to 5E5	1E5 to 5E5	1E5 to 5E5	1E5 to 5E5
**Isotope pattern min. score**	50%	50%	50%	50%
**Feature list methods > Gap filling > Same RT and m/z range gap filler**				
***m/z* tolerance**	0.0015 Da	0.0015 Da	Peak Finder, 20%	n/a
**Feature list methods > Filtering > Duplicate filter**				
**Filter mode**	New average	New average	New average	New average
***m/z* tolerance**	0.0015 Da	0.0015 Da	0.0015 Da	0.003 Da
**RT tolerance**	0.05 min.	0.05 min.	0.05 min.	0.10 min.
**Feature list methods > Filtering > Peak filter**				
**Height**	1E5 to 1E10	1E5 to 1E10	1E5 to 1E10	1E5 to 1E10
**No. of data points**	5 to 100	5 to 100	5 to 100	5 to 300
**Identification > Custom database search**				
***m/z* tolerance**	0.0015 Da	0.0015 Da	0.0015 Da	n/a
**RT tolerance**	0.02 min.	0.1 min.	0.1 min.	n/a
**Feature list methods > Filtering > Feature list rows filter**				
**Isotope pattern**	2	n/a	n/a	2
**Keep only with MS2**	Optional	n/a	n/a	checked

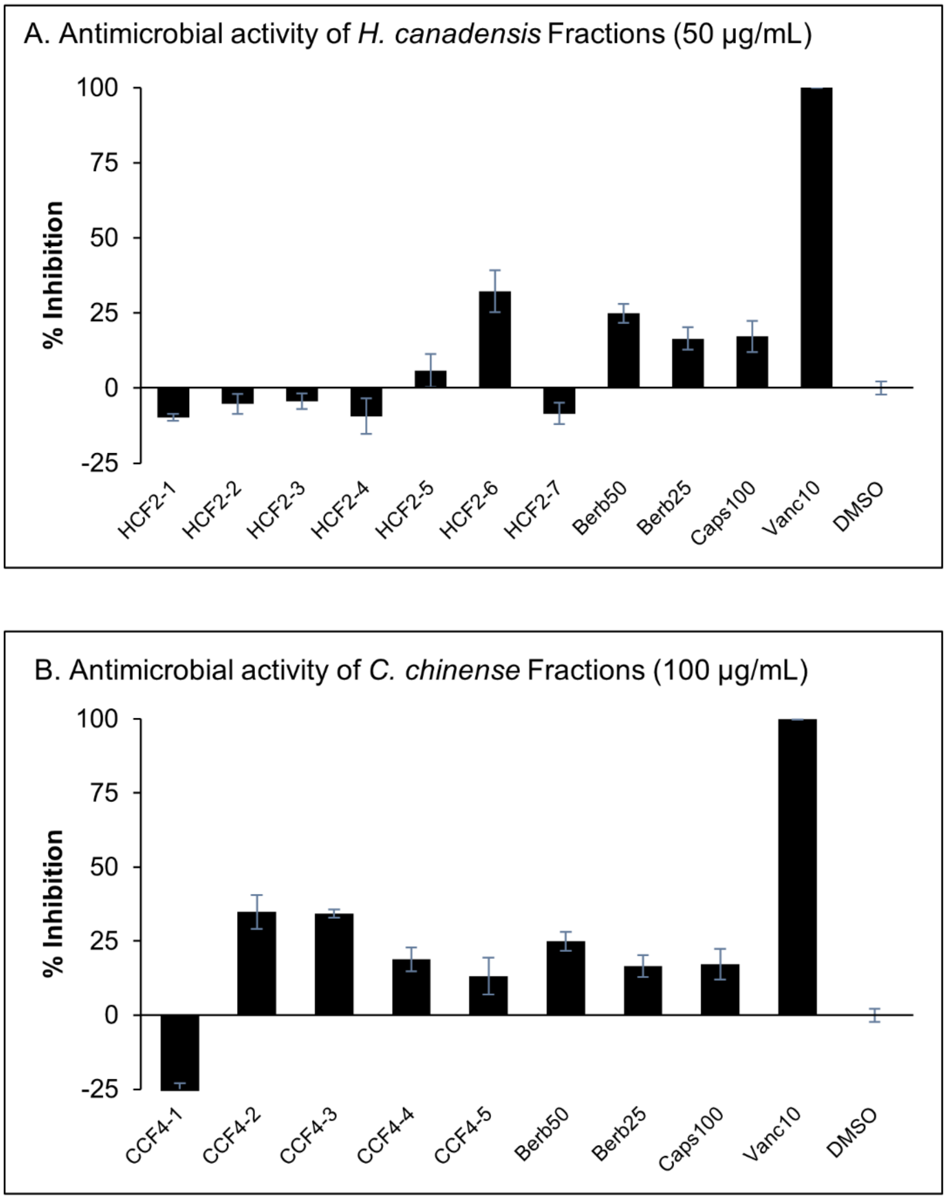

Figure S11.

- **A.** Antimicrobial activity of *Hydrastis canadensis* sub-fractions against *Staphylococcus aureus* strain SA1199 tested at 50 µg/mL. Vehicle (negative) control is 2% DMSO and vancomycin at 10 µg/mL, berberine at 50 and 25 µg/mL, and capsaicin at 100 µg/mL serve as positive controls. Values are reported as mean of biological replicates (triplicate wells) ± standard deviation.
- **B.** Antimicrobial activity of *Capsicum chinense* fractions at 100 µg/mL reported in comparison to the same controls used for panel (A).

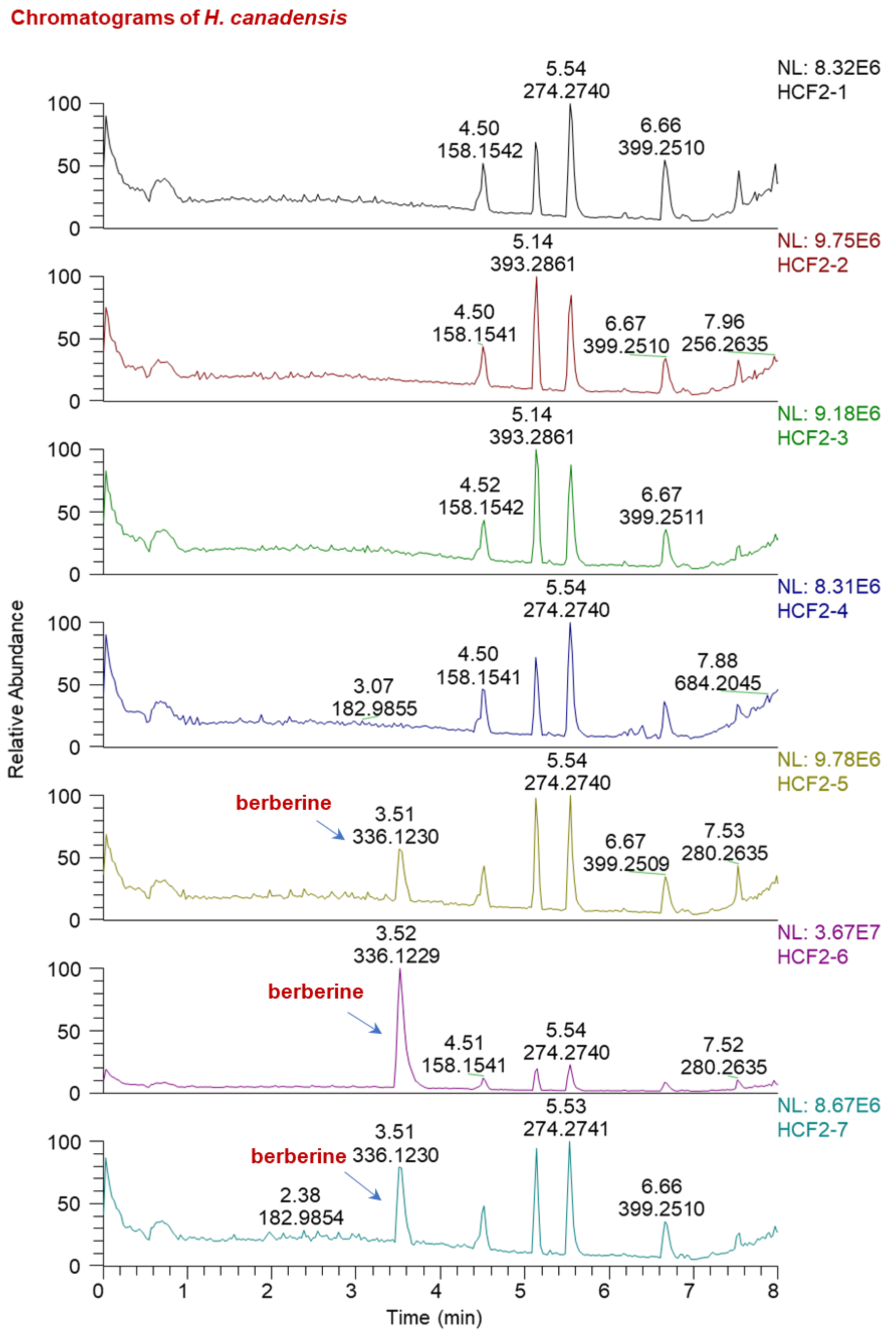

Figure S12. UPLC-MS base peak chromatograms obtained from *Hydrastis canadensis* sub-fractions (see Figure 6 in the main text for fractionation workflow).

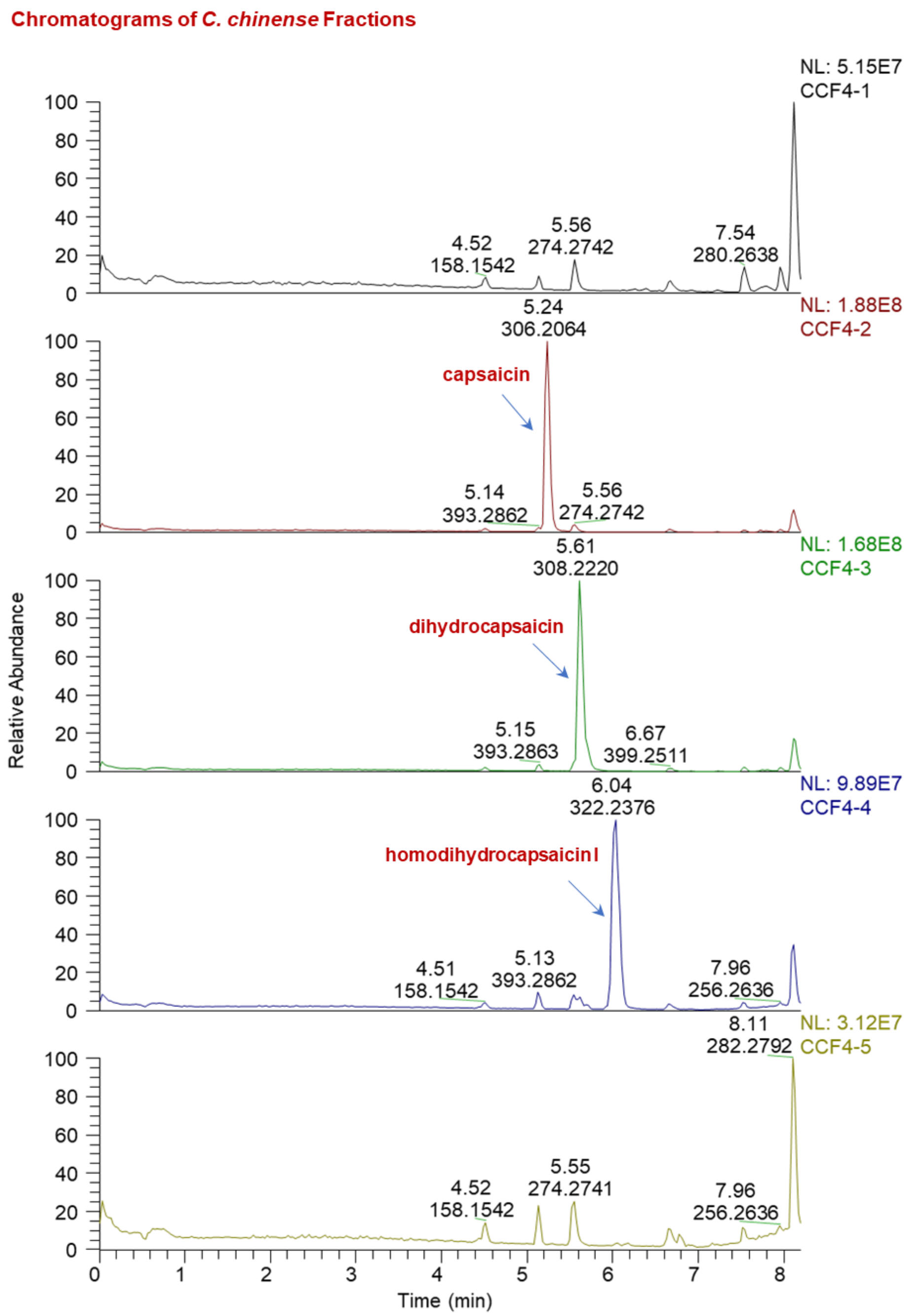

Figure S13. UPLC-MS base peak chromatograms obtained from *Capsicum chinense* sub-fractions (see Figure 6 in the main text for fractionation workflow).

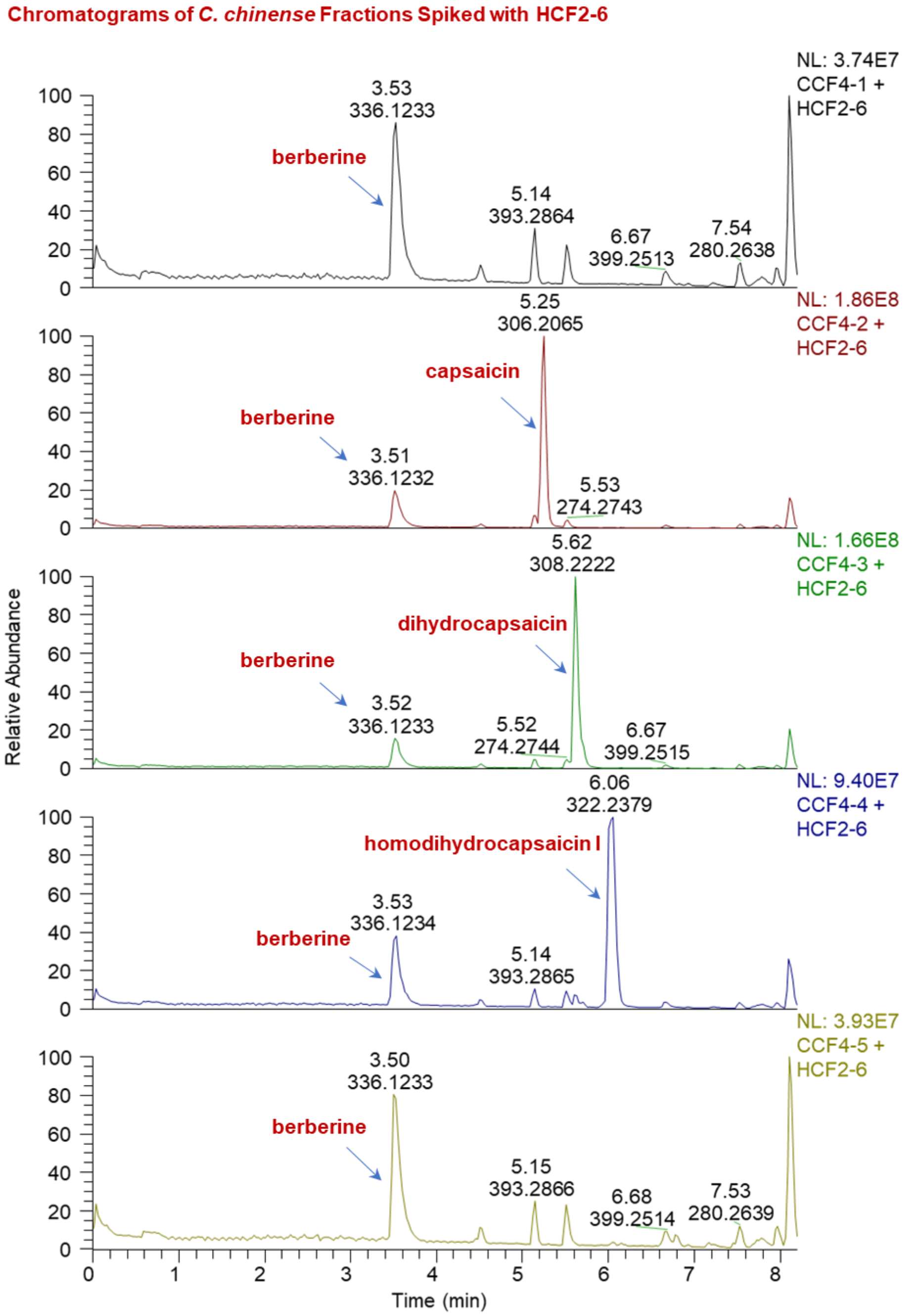

Figure S14. UPLC-MS base peak chromatograms obtained from mixtures of *Capsicum chinense* sub-fractions with a single *H. canadensis* sub-fraction.

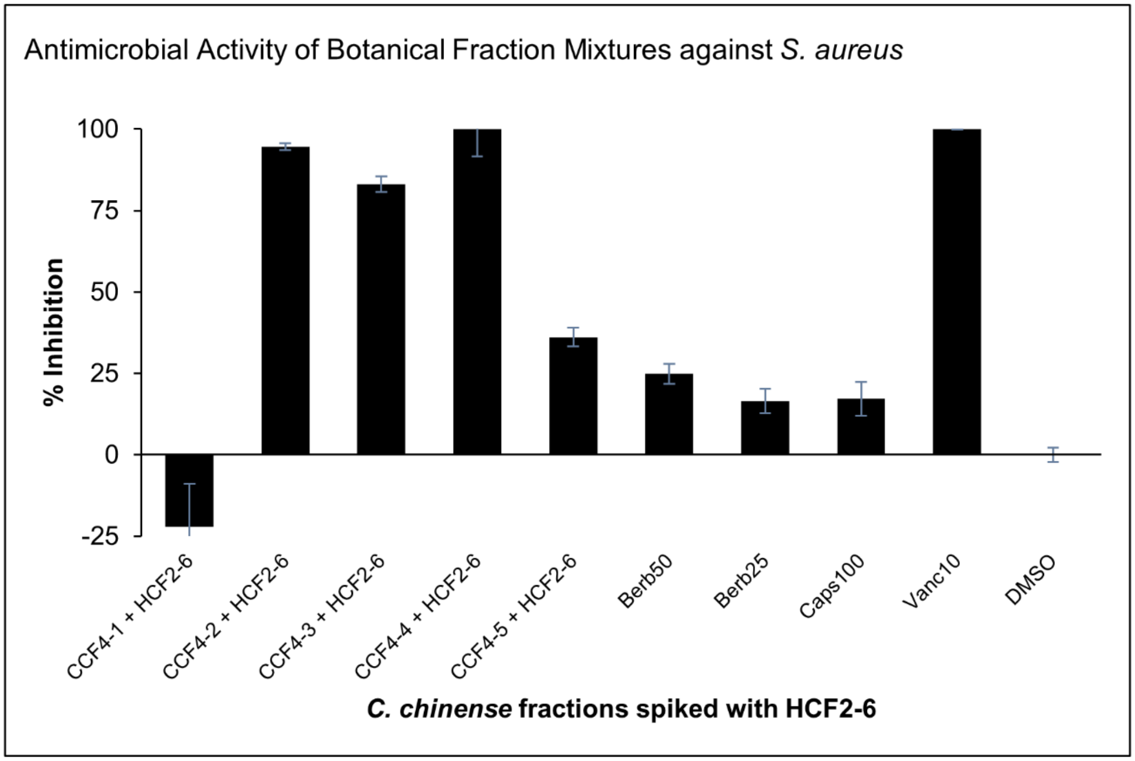

Figure S15. Antimicrobial activity for mixtures prepared from a single sub-fraction of an *Hydrastis canadensis* extract (HCF2-6) combined with a series of sub-fractions from a *C. chinense* extract (CCF4-1, CCF4-2, CCF4-3, CCF4-4, CCF4-5). Activity was evaluated against *Staphylococcus aureus* strain SA1199. Activities are reported as the mean % inhibition for triplicate wells with error bars representing standard deviation. Controls included isolated berberine at 50 and 25 µg/mL, isolated capsaicin at 100 µg/mL, vancomycin at 10 µg/mL, and vehicle (2% DMSO).

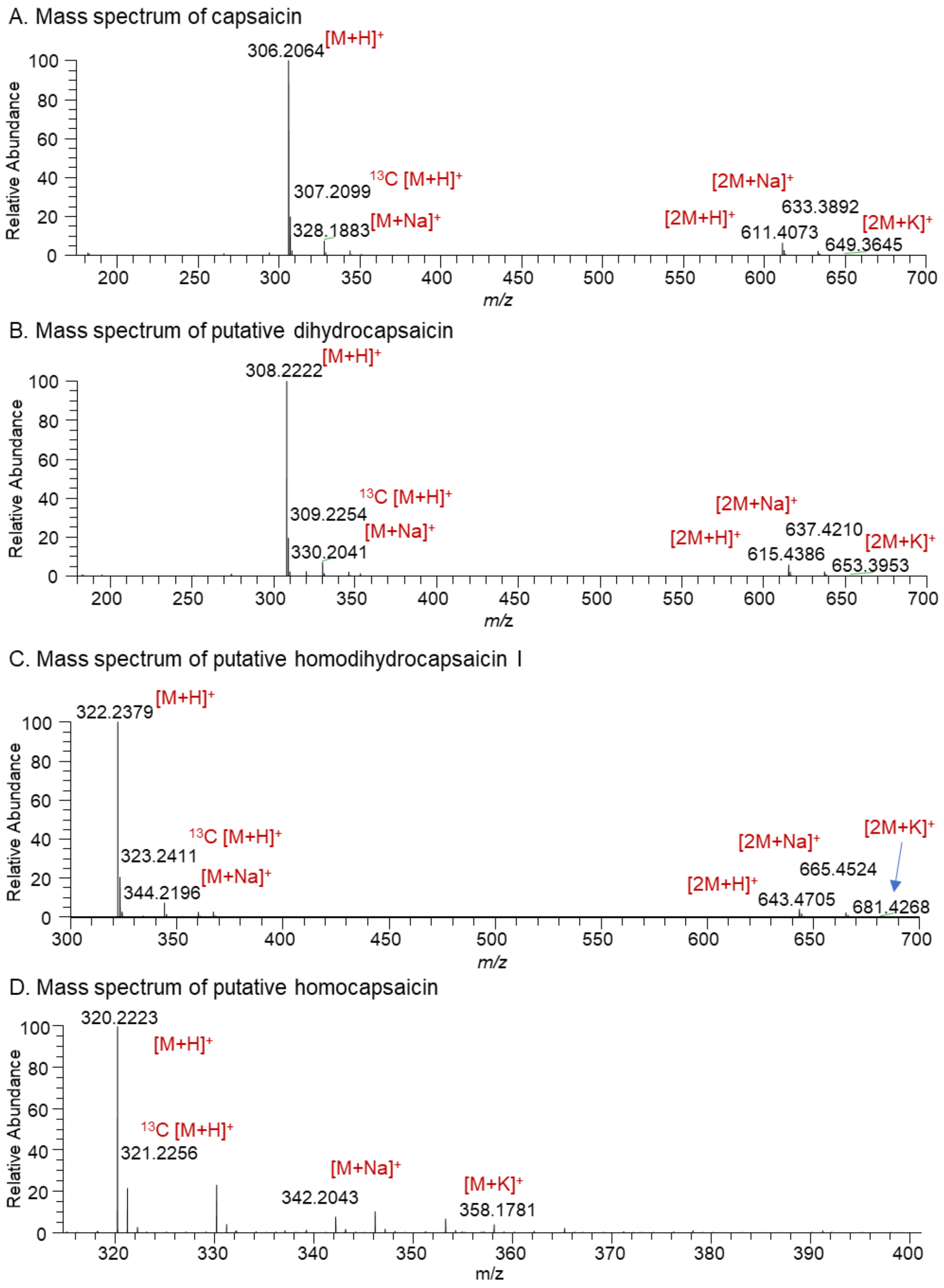

Figure S16. Mass spectra obtained from the *C. chinense* extract fractions assigned to capsaicin (retention time 5.24 min.), dihydrocapsaicin (retention time 5.61 min.), and homodihydrocapsaicin I (retention time 6.04 min). The identity of capsaicin (A) was confirmed by comparing retention time mass with a standard, while identities of dihydrocapsaicin (B), homodihydrocapsaicin I (C), and homocapsaicin (D) are putative only based on comparison of accurate mass with reported literature.

Table S10. PLS Modeling Information for *C. chinensis* fractions spiked with HCF2-6. 1

	**Classical Metabolomics**	**Interaction Metabolomics**
Object count	11	11
Variable count	22	132
Comp. retained	4	6
Comp. variance (x, y)	1 – 18.03%, 57.03%<br>2 – 15.17%, 24.6%<br>3 – 25.21%, 8.04%<br>4 – 10.56%, 1.81%	1 – 28.1%, 47.89%<br>2 – 28.7%, 19.58%<br>3 – 8.73%, 17.84%<br>4 – 21.24%, 2.78%<br>5 – 3.08%, 2.49%<br>6 – 1.38%, 6.93%
Monte Carlo validation threshold	0.364	0.273
RMSEP	24.68	22.18
R^2Y	0.92	0.98

Table S11. Mixture concentrations for *Capsicum chinense* fraction CCF4 and *Hydrastis canadensis* fraction HC-*aq* used to generate the Selectivity Ratio plot in Figure S17.

Mixture No.	CCF4 Concentration (µg/mL)	HC-*aq* Concentration (µg/mL)
1	3	40
2	3	1600
3	120	40
4	120	1600
5	60	800
6	30	400
7	30	1200
8	90	400
9	90	1200

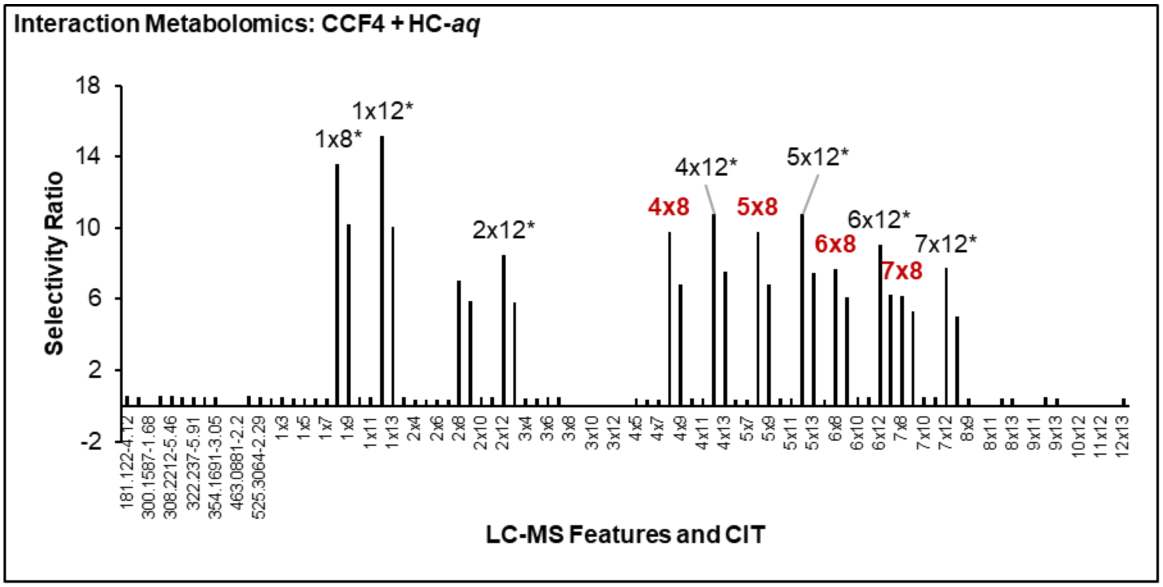

Feature	*m/z*	RT	Identity	Adduct Annotation
8	336.1220	3.19	berberine	M^+
4	306.2054	5.08	capsaicin	$[M+H]^+$
5	308.2212	5.46	dihydrocapsaicin	$[M+H]^+$
6	320.2210	5.52	homocapsaicin	$[M+H]^+$
7	322.2370	5.91	homodihydrocapsaicin I	$[M+H]^+$

Figure S17. Selectivity ratio data generated by combining *Hydrastis canadensis* aqueous partition (HC-*aq*) with the *Capsicum chinense* fraction CCF4 using the mixture concentrations indicated in **Table S11.** Only features shown in red are associated with berberine or capsaicinoids. *Interaction terms associated with unidentified features from *Hydrastis canadensis* or *Capsicum chinensis.*
